# The influence of an interfacial hBN layer on the fluorescence of an organic molecule

**DOI:** 10.3762/bjnano.11.149

**Published:** 2020-11-03

**Authors:** Christine Brülke, Oliver Bauer, Moritz M Sokolowski

**Affiliations:** 1 Institut für Physikalische und Theoretische Chemie, Universität Bonn, Wegelerstr. 12, 53115 Bonn

**Keywords:** decoupling, fluorescence, hexagonal boron nitride, 3,4,9,10-perylene tetracarboxylic dianhydride (PTCDA), Raman spectroscopy

## Abstract

We investigated the ability of a single layer of hexagonal boron nitride (hBN) to decouple the excited state of the organic molecule 3,4,9,10-perylene tetracarboxylic dianhydride (PTCDA) from the supporting Cu(111) surface by Raman and fluorescence (FL) spectroscopy. The Raman fingerprint-type spectrum of PTCDA served as a monitor for the presence of molecules on the surface. Several broad and weak FL lines between 18,150 and 18,450 cm^−1^ can be detected, already from the first monolayer onward. In contrast, FL from PTCDA on a bare Cu(111) surface is present only from the second PTCDA layer onward. Hence, a single layer of hBN decouples PTCDA from the metal substrate to an extent that a weak radiative FL decay of the optical excitation can occur. The different FL lines can be ascribed to different environments of the adsorption sites, namely molecules adsorbed at surface defects, in large ordered domains, and located in the second layer.

## Introduction

In recent years, two-dimensional materials (2DMs) have been established as a highly interesting field of studies, both in regard to their fundamental physical properties as well as their potential for technical applications [1]. Specifically, the use of 2DMs in layered heterostructures has been promoted [2–3]. Here, one challenge lies in the understanding of not only the processes in the individual materials, but also of those that occur at the interfaces between layers of different materials.

Advantageously, some 2DMs can be grown directly on metal substrates by chemical vapor deposition [2]. This is, for example, exploited when a 2DM interfacial layer is inserted between the metallic electrode and a functional organic layer of an organic electronic device, such as an organic light emitting diode [3]. The purpose of the interfacial layer is to achieve a separation or “decoupling” of the two adjacent layers. Here, the term decoupling refers to the spatial separation of the electronic states of the molecules and those of the underlying metal, which leads to unperturbed molecular properties [4]. A scientifically relevant question is to which extent decoupling of the organic molecules from a metal electrode is achieved when a 2DM layer in the limit of a single interfacial layer, for example, a monolayer of hexagonal boron nitride (hBN), is used.

Such a decoupling is achieved when the wave functions of the metal are spatially separated from those of the molecule leading to a reduced overlap. The overlap of molecular and metallic wave functions has, in particular, an impact on excited molecular states. For fluorescent molecules, this is observed as a strongly reduced quantum yield of the fluorescence (FL) due to non-radiant decay processes via the metal states. This phenomenon is generally referred to as “quenching” [5]. When the decoupling is not efficient, a fast and non-radiative decay of the excitation of the molecule via the metallic states prevails. Therefore, the probing of the FL of a molecule located at the outer surface of a 2DM layer grown on a metal characterizes the degree of electronic coupling of the molecular and metallic states.

**Figure 1:**
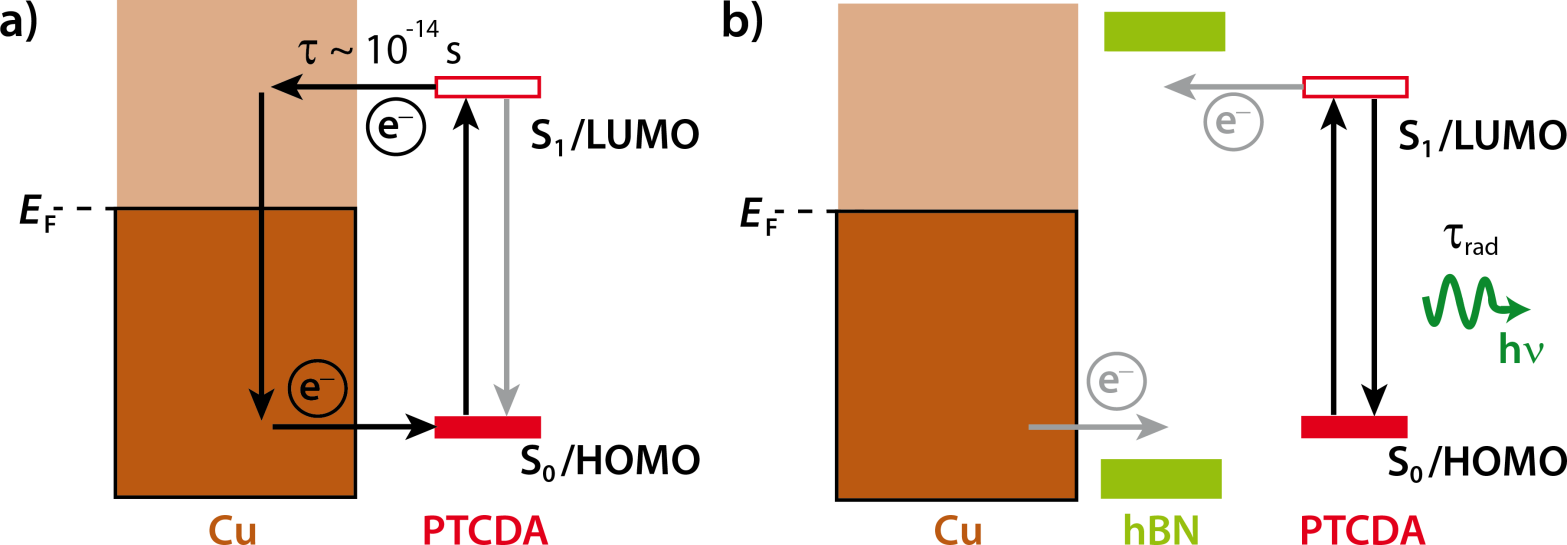
(a) Schematic diagram of the radiative and non-radiative decay processes of an optical excitation of a PTCDA molecule at the interface to a Cu surface via charge transfer. Metallic and molecular electronic states are indicated in brown and red, respectively. The radiative decay of the excitation (gray arrow) is quenched. (b) A decoupling layer of hBN (green) suppresses the overlap of the Cu and PTCDA wave functions. This reduces the charge transfer such that the excitation decays under emission of a photon (green arrow).

For completeness, we note that quenching of an electronic excitation of a molecule in the first layers on a metal surface can be the result of interfacial charge transfer (CT) [5] or of energy transfer [6]. Here, CT is in our focus since energy transfer, although additionally present, varies less abruptly on the scale of single layers [7]. A simple energy diagram of the CT process of a fluorescent molecule across interfaces is depicted in Figure 1. As an example, we use the sample system of this work, namely, 3,4,9,10-perylene tetracarboxylic dianhydride (PTCDA) on a layer of hBN on Cu(111). Here, we consider an S_1_ excitation which involves mainly a HOMO/LUMO (highest occupied and lowest unoccupied molecular orbital) electronic excitation. Rapid CT leads to a delocalization of the excited electron from the LUMO into unoccupied metallic states and/or a filling of the HOMO by an electron from the metal. We note that the HOMO and LUMO for the chemisorbed molecule differ from those of the gas phase molecule. Thus, the LUMO that is drawn in Figure 1a is not identical to the LUMO in the gas phase. For a second molecular layer, even without significant overlap of the wave functions of metal and molecule, quenching is also possible, because the CT may occur from the second layer to the metal via states in the first layer [5].

In principle, the hybridization of molecular and metallic states and the dynamical CT in the excited state can be probed by photoemission spectroscopy (PES). In particular, core hole clock spectroscopy has been used to measure the time constant of dynamical CT in the valence band states as a function of the lifetime of the core hole, which is typically of the order of 10^−14^ s [8–9]. However, this technique is insensitive to a CT process of a considerably longer time constant. A technique for detecting CT processes with a longer time constant is FL spectroscopy, in which the CT process competes with the radiative fluorescent decay of the excitation. It constitutes an alternative and non-radiative decay channel for the excited state. Typically, the FL lifetime of the excited state S_1_ is of the order of ca. 10^−9^ s [5]. Hence, a CT process with a time constant that is not significantly larger will reduce the FL yield (see Figure 1). Thus, even a very small overlap of states becomes evident in the experiment as it causes a reduction of the FL yield (i.e., quenching). The degree of quenching may vary and, hence, lead to different branching ratios between the radiative and non-radiative channels. In order to obtain high FL yields of molecules on metallic substrates, thin interfacial films of considerable thickness (5–10 monolayers) of alkali halides have been used in our lab [10]. In contrast, experiments on the light emission from molecules induced by scanning tunneling microscopy (STM-LE) required thin alkali halide films of two monolayers thickness in order to support tunneling [11–13].

A single layer or films of hBN are attractive for decoupling a molecule from an underlying metal substrate as hBN exhibits a wide bandgap of 5.9 eV [14]. Perspectively, it could also provide a substrate for STM-LE experiments. Furthermore, it is of interest due to its mechanical [15], chemical [16], and thermal [17] stability, the easy synthesis of hBN monolayers on Cu foils for usage in devices [18], and, finally, the wide structural variety of hBN monolayers depending on the underlying metal substrate [19].

To investigate the decoupling of an organic molecule from a metal substrate by a monolayer of hBN we chose PTCDA and the Cu(111) surface. PTCDA serves as a planar model molecule for investigations of organic layers on surfaces [20–23]. Monolayers of hBN on Cu(111) have been investigated by several groups and are, hence, rather well understood [24–29]. In particular, we found that hBN on Cu(111) forms a flat layer at a relatively large vertical distance from the Cu(111) top layer of 3.24 Å using an X-ray standing-waves analysis [30]. This large distance is, in principle, in agreement with the results reported independently by Schwarz and co-workers [31]. Large distances of the molecule with respect to the hBN and the metal interface are expected to be beneficial for the decoupling because the metal wave functions decrease exponentially into the vacuum.

We have previously shown that the bonding situation between PTCDA and hBN/Cu(111) is weak and physisorptive [32] as opposed to the chemisorptive bond between PTCDA and Cu(111) [33]. Ultraviolet photoelectron spectroscopy (UPS) experiments showed that on the Cu(111) surface the chemical bonding leads to a filling of the LUMO [33]. In contrast, on hBN/Cu(111), the differential energies of the PTCDA orbitals remain unaltered in comparison to those of PTCDA in the gas phase, which points to a more physisorptive bonding to the hBN/Cu(111) surface [32]. The HOMO of PTCDA is found at ca. 2.6 eV [32]. Hence, we can expect that both the LUMO and the HOMO are placed within the bandgap of hBN, as indicated in Figure 1b. This is in agreement with the findings by Martínez-Galera et al. for PTCDA/hBN/Rh(110) [34]. From scanning tunneling spectroscopy (STS) experiments, the authors concluded that the coupling is only weak. They deduced further that the CT (in the ground state) between molecule and substrate, if present at all, is small. These differences between PTCDA/hBN/Cu(111) and PTCDA/Cu(111) are also mirrored by their vertical molecular structures. The hybridization of the electronic states of PTCDA and Cu leads to a distortion of the molecule where the oxygen atoms are pushed away from the substrate and out of the molecular plane [35]. On hBN/Cu(111), the molecule remains essentially flat and at a very large vertical distance from the hBN layer of 3.37 Å [32]. In contrast, on Cu(111) the vertical distance of the perylene core from the Cu(111) surface is only 2.66 Å [35]. This again points to a difference in the bonding character on the two surfaces.

Several studies have probed the influence of the adsorption on metal-supported hBN layers on the electronic structure of large organic molecules, namely their frontier orbitals, by PES [36] or STS [37–38]. However, to the best of our knowledge, there have been no studies on the FL of monolayers of molecules on metal-supported hBN layers, yet. Kerfoot et al. [22] studied the FL of PTCDA and perylene-3,4,9,10-tetracarboxylic-3,4,9,10-diimide (PTCDI) on an exfoliated hBN monolayer that was transferred onto SiO_2_. Forker et al. [23] investigated the optical absorption properties of PTCDA on hBN/Rh(111) and hBN/Pt(111).

Here, we report a direct comparison of FL spectra of PTCDA/hBN/Cu(111) and PTCDA/Cu(111), which allows for a relative determination of the efficiency of the hBN layer to decouple the excited states of PTCDA from Cu(111). For PTCDA on Ag(111) and Au(111) [39], it has been shown that FL can only be observed from the second and third molecular layer onward. The excitation of the first layers is completely quenched by the metal substrates as described above. In UPS experiments, a partial filling of the LUMO of PTCDA was found on Ag(111), but not on Au(111) [33]. Thus, the quenching on Ag(111) is directly understood by the static charge transfer seen in UPS. The quenching on Au(111), not as evident from UPS, demonstrates the sensitivity of FL spectroscopy to an overlap of wave functions of excited states. Accordingly, the same behavior as on Ag(111) can be expected on Cu(111), where a filling of the LUMO has been found, too [33]. In addition, for multilayer PTCDA films we can compare the first, interfacial PTCDA layer with a hBN layer regarding their abilities to decouple the next PTCDA layer from the Cu(111) substrate.

In this contribution, we will discuss Raman modes and several different FL lines of PTCDA that were observed on both hBN/Cu(111) and Cu(111). For an effective comparison, the structures and the growth modes of the PTCDA layers are relevant. Details of the structural investigation of the two surfaces, including low-energy electron diffraction (LEED) patterns, are given in Appendix A. Since the interpretation of the optical data requires this knowledge, we summarize some details ahead here. PTCDA forms ordered structures and follows a layer-by-layer growth for at least the first three layers on Cu(111) and the first two layers on hBN/Cu(111). We are able to distinguish PTCDA/hBN/Cu(111) from PTCDA/Cu(111), as well as the monolayer and multilayers of PTCDA on Cu(111) and hBN/Cu(111) by differences in the respective LEED patterns.

## Experimental

All experiments were carried out in an ultrahigh-vacuum chamber with a base pressure of 2.3 × 10^−10^ mbar. The Cu(111) sample could be heated up to 1100 K via a tungsten filament and electron bombardment and cooled down to 20 K by using liquid helium.

The hBN layer was grown by dosing the precursor borazine [(HBNH)_3_] into the chamber while the sample was held at 1010 K. Borazine was purchased from Katchem spol. s r. o., Czech Republic (http://www.katchem.cz). It was kept in a glass tube connected to the chamber via a dosing valve at constantly −5 °C. Prior to the hBN preparation, it was cleaned by three cycles of freezing the liquid borazine using liquid N_2_ and pumping away the gas phase above the frozen borazine. The composition of the gas phase in the borazine source was monitored by a quadrupole mass spectrometer (QMS). It was considered suitable for hBN preparation when the signals for H_2_ (a product of the decomposition of borazine that is known to occur even when stored at low temperatures, *m*/*z* 2) and borazine (*m*/*z* 80) showed a ratio of approximately 1:1.

The clean Cu(111) surface was prepared by consecutive steps of sputtering for 30 min with Ar^+^ ions (1000 eV, 4 μA) and annealing at 1010 K for 30 min. After the last sputtering cycle, the Cu(111) sample was heated to 1010 K and ca. 2000 L borazine were dosed (1.5 × 10^−6^ mbar via the background for 30 min) onto the sample held at 1010 K. After stopping the borazine dosing, the sample was cooled down with 1 K·s^−1^. The structural quality of the bare Cu(111) surface, the hBN layers, and the PTCDA layers was checked by LEED. We used a SPA-LEED instrument as described in [30]. An additional annealing step between the last sputter cycle and the borazine deposition was omitted here in order to prevent segregation of chemical impurities from the Cu bulk to the surface, which we otherwise occasionally observed as additional LEED spots. The criteria for a good hBN layer were a sharp continuous ring of intensity with a radius that is 2% larger than the distance of the first-order Cu spots from the specular spot and the appearance of a clear star-like pattern of satellites around the specular spot caused by multiple electron scattering as reported in [30].

PTCDA was evaporated from a glass crucible. The molecular flux was also monitored by the QMS at *m*/*z* 392, which corresponds to the mass of the non-fractured PTCDA molecule. The flux was typically one monolayer per minute. During deposition, the sample was held at a constant temperature. PTCDA layers on Cu(111) were prepared by keeping the sample at a temperature of either 20 or 300 K during deposition. PTCDA layers on hBN/Cu(111) were prepared by deposition at a sample temperature of 20 K and subsequent annealing. The sample was annealed in iterative steps of heating, holding at a constant temperature for 3 min, and cooling down again to 20 K for a measurement. The annealing temperatures were in the range of 100–400 K and are specified below. In the following, one monolayer (ML) of PTCDA refers to a single layer of close-packed, flat-lying PTCDA molecules. After every experiment, the exact coverage was determined by a thermally programmed desorption (TPD) experiment with a margin of error of ±0.05 ML. The calibration for PTCDA/hBN/Cu(111) was derived from the TPD data shown in [32]. Since the packing densities of PTCDA on hBN/Cu(111) and on Cu(111) are within a few percent of each other (see Appendix A), this calibration is also valid for PTCDA/Cu(111) within the noted margin of error.

For the optical experiments, the sample was transferred into a glass tube, standing out from the end of the chamber. In the glass tube, the sample was illuminated by a focused laser beam at an incident angle of approximately 45° with respect to the surface normal. The diameter of the laser spot on the sample was about 0.5 mm. The fluorescence and Raman scattered light from the sample was collected and parallelized by an achromatic lens and focused by a second achromatic lens onto the entrance slit of the spectrometer. The spectrometer (Acton, Spectra Pro 2300i, *f* = 0.3 m) was operated with three different gratings (300, 600, and 1200 grooves per millimeter, yielding a resolution of 48, 24, and 12 cm^−1^, respectively). It was equipped with a nitrogen-cooled CCD camera, operated at −110 °C. In order to block external stray light from entering the spectrometer, the glass tube, the lenses and the entrance slit of the spectrometer were enclosed by a black box. If not specified otherwise, we used an optically pumped cw semiconductor laser (Coherent Sapphire LP UBB CDRH) with a wavelength of 458 nm (photon energy of 2.698 eV or 21,816 cm^−1^) and *P* = 50 mW. To block the laser light from entering the spectrometer, a long-pass filter (cut-off at 475 nm) was positioned in front of the entrance slit of the spectrometer. All spectra were recorded at a sample temperature of 20 K with an exposure time of 2 s, 50 accumulations, and a slit width of the spectrometer of 0.1 mm.

## Results and Discussion

### 1 Overview of the spectra

**Figure 2:**
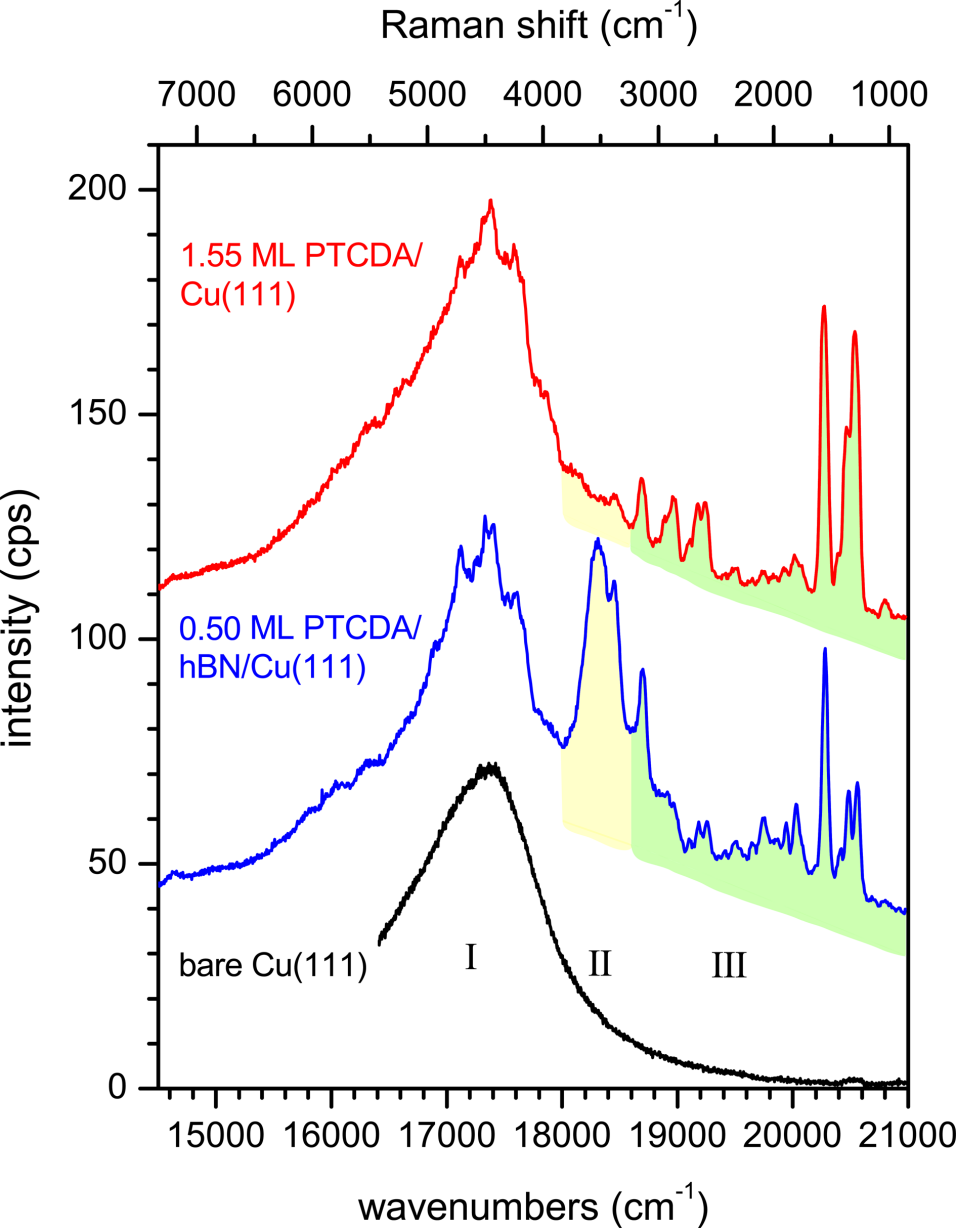
Overview spectra of 1.55 ML PTCDA on Cu(111) (red), of 0.50 ML PTCDA on hBN/Cu(111) (blue), and of the clean Cu(111) surface (black). For preparation details, see text. We distinguish three regions I, II, and III. The sharp lines marked in green are Raman modes (region III). The features marked in yellow are assigned to fluorescence (region II). The broadest feature on the low-energy side is due to defect luminescence of the Cu substrate (region I). All spectra were measured at 20 K using a grating with 300 grooves per millimeter. The spectra are shifted vertically for clarity.

Figure 2 shows overview spectra of 1.55 ML PTCDA on Cu(111) (red spectrum) and 0.50 ML PTCDA on hBN/Cu(111) (blue spectrum). PTCDA on Cu(111) was deposited at a sample temperature of 300 K and the LEED pattern confirmed the formation of a second layer (see Appendix A). PTCDA on hBN/Cu(111) was deposited at a sample temperature of 20 K and subsequently annealed at 300 K. The FL spectrum shown here is identical to the FL spectrum of PTCDA deposited at a sample temperature of 300 K, which showed the LEED pattern in Figure 8b (see below in Appendix A), revealing an ordered structure. Hence, we conclude that the blue FL spectrum shown in Figure 2, which is, as it will become clear, relevant for the comparison with PTCDA/Cu(111), also stems from an ordered PTCDA layer on hBN/Cu(111).

For the discussion of the spectra, we consider three regions (I–III). At first glance, two of these regions appear qualitatively rather equal for both substrates: On the low-energy side below 18,000 cm^−1^ (region I) a broad luminescence can be observed and on the high-energy side above 18,600 cm^−1^ (region III), there is a set of sharp peaks. However, the two spectra differ significantly between 18,000 and 18,600 cm^−1^ (region II) due to broad FL peaks (highlighted in yellow) present for hBN/Cu(111), but absent for PTCDA/Cu(111).

We tentatively assign the broad peak in region I to a radiative decay of interband transitions from the Cu substrate, as it can also be observed on the clean substrates (Cu(111) and hBN/Cu(111)) before PTCDA deposition when all other features are absent (see Figure 2, black spectrum). An enhancement of radiative interband transitions has been reported for Cu nanoparticles [40]. We thus speculate that surface defects (protrusions) play a role here. This is in agreement with our observation that the intensity of this “defect luminescence” in region I depends on the exact position of the spot of the excitation light on the sample. It will not be in the focus of this work. The sharp peaks in region III are identified as Raman lines from PTCDA (highlighted in green). The Raman lines were additionally identified by using a dye laser with tunable wavelength [20] (497–507 nm). These peaks shift according to the wavelength of the laser (see Appendix B). Notably, some Raman peaks are superposed in the region of the defect luminescence of the Cu(111) surface (region I), too.

### 2 Raman modes

First, we will discuss the Raman lines. The peaks in the spectrum between 21,000 and 18,600 cm^−1^ in Figure 2 exhibit Raman shifts that correspond to the energies of the vibrational modes of PTCDA adsorbed on surfaces observed before [41–42]. The vibronic modes of PTCDA that can be observed in Raman spectroscopy are A_g_, B_1g_, B_2g_, and B_3g_ modes, with the most prominent modes being A_g_ modes between 1,250 and 1,650 cm^−1^ [41–43]. The spectral positions of most of these modes are about constant for PTCDA adsorbed on different substrates [41] or for different film thicknesses [42]. An interesting exception was observed for PTCDA on Ag(111) [42] for the breathing mode of the central carbon ring at ca. 1,300 cm^−1^. In the following, we will refer to it as ring breathing (RB) mode. On Ag(111), two different adsorption states of PTCDA were observed. Both states are bonded chemisorptively to the surface [44]. The RB mode of PTCDA deposited at a low temperature (LT) of 180 K exhibits a Raman shift of 1,310 cm^−1^. This is higher in energy by 13 cm^−1^ compared to the Raman shift (1,297 cm^−1^) for the RB mode of a layer at room temperature (RT) [42]. The special role of this RB mode will be discussed in further detail below.

#### 2.1 Surface-enhanced Raman scattering

The fact that the Raman modes of a small quantity of molecules can be observed here at all is attributed to surface-enhanced Raman scattering (SERS) [45]. This effect is most commonly observed on rough surfaces of noble metals [45] or at metal nanostructures [46], and it is utilized in surface-enhanced Raman spectroscopy [47]. There are two explanations for it, namely, a chemical mechanism and an electromagnetic mechanism, which is thought to be the dominant contribution to the enhancement. The chemical mechanism is related to the specific chemical surface bonding of the investigated system. At its heart, a CT between the molecule and the substrate occurs due to the chemisorptive bonding, which leads to a change in the polarizability of the molecule and thus to an enhancement of the Raman signal. It is also possible that electronic excitations of the adsorbed molecule allow for a resonance Raman effect, which causes an additional enhancement [46]. According to the electromagnetic mechanism, on a rough surface, surface plasmon polaritons (SPPs) can also be excited by the incident light. The surface plasmons are located in the vicinity of surface defects, such as protrusions. The field enhancement at these defects leads to an enhancement of the Raman scattering [48]. Subsequently, the scattered light can be enhanced in the same manner. The electromagnetic mechanism may be responsible for an enhancement of the signal by a factor of 10^5^–10^6^ [48]. The contribution of the chemical mechanism is generally much smaller, causing an enhancement by a factor of not more than 10^2^–10^3^ [46].

Recently, hBN has gained interest as a SERS substrate [49]. In a comparative study on 2DMs on SiO_2_ it was shown that hBN had an enhancement effect on the Raman modes of adsorbed copper phthalocyanine molecules [50]. The effect was explained by the polar character of the B–N bonds, which induced a dipole in the adsorbed molecule. The resulting interfacial dipole–dipole interactions are thought to have a similar effect on the polarizability of the adsorbed molecule as a CT.

Regarding the Raman enhancement effect of a noble metal surface, we mention a recent study by Stallberg et al. [39], which investigated optical spectra of PTCDA on Ag(111) and Au(111). They found Raman modes of PTCDA on the Au(111) surface, but not on the Ag(111) surface. This observation was discussed in view of the different energies of the SPPs of the two surfaces. Stallberg et al. used photon energies of 2.37 eV on Au(111) and 2.43 eV on Ag(111) and concluded that only the SPP of Au(111) located at 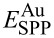 = 2.5 eV can resonantly interact with the incident light, leading to an enhancement of the Raman modes. The SPP on the Ag(111) surface has an energy of 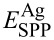 = 3.7 eV. Hence, a resonance was considered less probable, yielding no enhancement of Raman modes. This model should evidently encompass that the coupling to the SPPs requires a rough surface or local protrusions on the surface due to defects that break the translational symmetry. For comparison, we note that the energy of the SPP of Cu(111), which is calculated from the condition 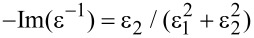 [51] using the dielectric functions given in [52], is obtained at a value of 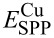 = 2.3 eV. Our excitation energy was 2.698 eV. From our experiment we have indeed evidence that SERS is related to surface defects. This will be discussed in Section 2.3.

#### 2.2 Raman modes of PTCDA on hBN/Cu(111) and Cu(111)

**Figure 3:**
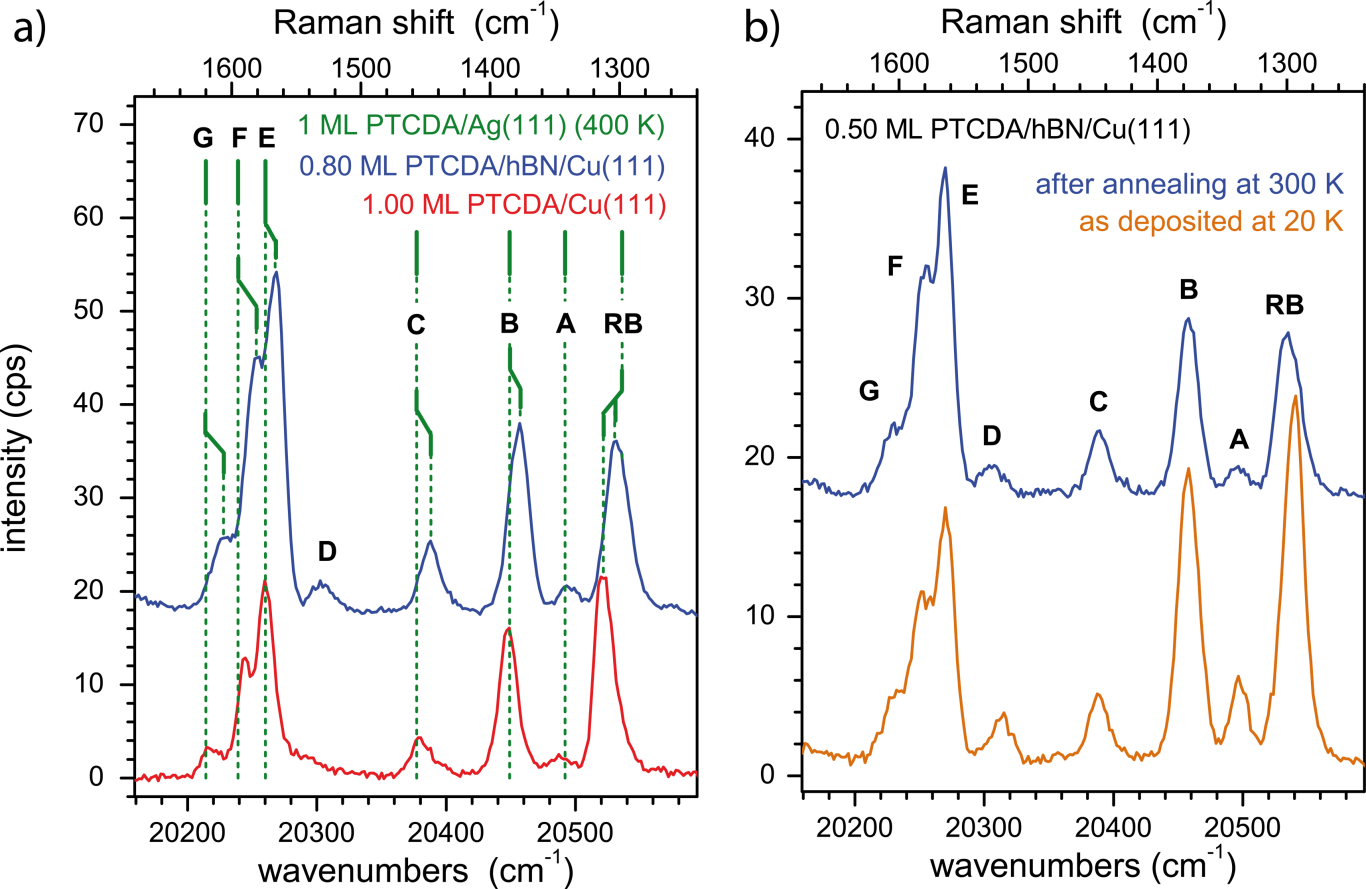
Zoom-in on the high-energy region (III) of the spectra. (a) 0.80 ML PTCDA/hBN/Cu(111) after deposition at 20 K and subsequent annealing at 300 K (blue) and 1.00 ML PTCDA/Cu(111) after deposition at 300 K (red). The positions of the Raman modes of 1 ML PTCDA/Ag(111) [53] are indicated in green. (b) 0.50 ML PTCDA/hBN/Cu(111) after deposition at 20 K (orange) and after subsequent annealing at 300 K (blue). All spectra were measured at 20 K using a grating with 1200 grooves per millimeter. Labels of the peaks refer to Table 1, the peaks H–K are not shown here.

Figure 3a and Figure 3b show region III of the spectra of PTCDA on hBN/Cu(111) and Cu(111) for Raman shifts of 1,240 to 1,660 cm^−1^ in detail. All Raman shifts are also summarized in Table 1. Some values are given as averages for different coverages as no trends as a function of the coverage were found (see caption). First, we discuss spectra of PTCDA on hBN/Cu(111) and Cu(111), which were both stable under annealing at 300 K (cf. Figure 3a) and, hence, represent the final state. Kinetic effects do not play a role here. In Section 2.2.2, we focus on temperature-dependent effects (cf. Figure 3b).

**Table 1:** Raman shifts (in cm^−1^) of PTCDA on hBN/Cu(111) and Cu(111), prepared at sample temperatures *T*_S_ (for details on the preparation, see text). Data were measured at 20 K. The values for PTCDA/hBN/Cu(111) are averaged for different film thicknesses (eight datasets with coverages between 0.05 and 0.80 ML for both 20 and 300 K). The error margins given are the standard deviation. The values for PTCDA/Cu(111) refer to coverages of 1.20 ML for *T*_S_ = 20 K and 1.00 ML for *T*_S_ = 300 K. For comparison, the Raman shifts of 1 ML PTCDA/Ag(111) measured by Schneider et al. [53], the vibrational energies of PTCDA/KCl/Ag(100) measured by Paulheim et al. in FL experiments [54], and the Raman shifts of the PTCDA single crystal measured by Tenne et al. [55] are listed. The ring breathing (RB) mode of the central carbon ring of PTCDA is the only mode that changes as a function of film thickness. The values for the RB mode of multilayers (RB_multi_) refer to 5.10 ML and 8.20 ML PTCDA/hBN/Cu(111) (averaged values), 3.44 ML PTCDA/Cu(111) for *T*_S_ = 20 K, 2.55 ML PTCDA/Cu(111) for *T*_S_ = 300 K, and 60 ML PTCDA/Ag(111) [53]. The modes of PTCDA/Cu(111) with *T*_S_ = 20 K are intrinsically very small and often not observed. Those modes are listed as “n.o.”.

mode	hBN/Cu(111)	hBN/Cu(111)	Cu(111)	Cu(111)	Ag(111) [53]	KCl [54]	PTCDA single crystal [55]^a^
*T*_S_	20 K	300 K	20 K	300 K	400 K	*<*20 K	

RB_mono_	1296.7 ± 1.6	1301.2 ± 1.7	1304.0	1312.9	1298	1288	—
RB_multi_	1301.8 ± 3.8	1309.8 ± 3.1	1300.0	1309.5	1309	—	1302.3
A	1339.4 ± 1.5	1339.2 ± 1.2	n.o.	1346.5	1342	1332	1335.0
B	1378.0 ± 1.0	1377.5 ± 1.5	1383.8	1386.0	1385	1368	1375.4/1383.6
C	1447.8 ± 1.5	1447.4 ± 1.7	n.o.	1454.6	1457	1446	1451.0
D	1522.4 ± 2.1	1529.4 ± 0.7	n.o.	n.o.	—	1523	—
E	1566.6 ± 1.9	1567.9 ± 2.5	1571.4	1574.0	1574	1564	1570.6
F	1582.7 ± 3.7	1582.3 ± 2.0	n.o.	1589.6	1595	1584	1589.1
G	1606.7 ± 1.7	1607.3 ± 2.0	n.o.	1616.2	1620	—	1615.0
H	1674.3 ± 0.7	1673.3 ± 2.4	n.o.	n.o.	—	—	—
I	1757.8 ± 1.8	1758.0 ± 1.7	n.o.	1764.3	—	—	—
J	1795.0 ± 1.3	1796.2 ± 2.2	n.o.	n.o.	—	—	1783.0
K	1820.4 ± 1.2	1825.9 ± 1.3	n.o.	1833.7	—	—	—

^a^Only the upper Davydov components are listed.

**2.2.1 The final state – 300 K spectra:** Figure 3a shows Raman modes of PTCDA/hBN/Cu(111) after deposition at 20 K and subsequent annealing at 300 K (blue), and of PTCDA/Cu(111) after deposition at 300 K (red). The positions of the Raman modes of 1 ML PTCDA/Ag(111) measured by Schneider and Wagner [42,53], which was deposited at a sample temperature of 400 K, are indicated by green vertical bars for comparison. It is apparent from Figure 3a that the modes of PTCDA/hBN/Cu(111) are systematically shifted to smaller energies by about 7 cm^−1^ compared to PTCDA/Cu(111). In contrast, the modes of PTCDA/Ag(111) [53] agree well with the modes of PTCDA/Cu(111) within 0.3%. An exception is the RB mode. This mode will be discussed separately below. The shift of the other modes to higher vibrational energies seen in comparison of hBN/Cu(111) and the metal surfaces Cu(111) and Ag(111) can be linked to the different bonding of PTCDA to the surfaces. In UPS experiments [33], a chemisorptive interaction of the metal surfaces with the PTCDA molecule was found, leading to a (partial) filling of the former LUMO on Ag(111) and Cu(111). On PTCDA/hBN/Cu(111), no such chemisorptive bonding was observed in UPS [32]. We suppose that the energy of the vibrational modes recorded here are influenced by molecule–substrate interactions. The chemisorptive bond to the metal surface makes the intermolecular bonds harder, which causes the respective vibrational modes to increase in energy. For Raman shifts below 1,500 cm^−1^, this interpretation is also supported by the closer agreement of the Raman modes on hBN/Cu(111) with those measured for PTCDA single crystals [55] (see Table 1). However, for modes above 1,570 cm^−1^, the situation is reversed and not yet understood.

As mentioned before, the significance of the RB mode has been reported for PTCDA/Ag(111) [53]. The energy of this mode increases for PTCDA on different substrates, going from KCl films on Ag(100) (1,288 cm^−1^, derived from FL spectra) [54], to Ag(111) (1,298 cm^−1^) [53], hBN/Cu(111) (1,301 cm^−1^, derived from FL spectra), and Cu(111) (1,313 cm^−1^, derived from FL spectra), contrary to the other modes. This trend neither conforms with the strength of the (chemisorptive) bond to the substrate surface [33], nor with the bonding distance, or the amount of molecular distortion [32,35,56–57]. However, in some way it reflects the change in the distortion motif of the PTCDA molecule. On KCl/Ag(100), the PTCDA molecule is bend like an arch with all of the oxygen atoms pulled towards the surface [57]. On Ag(111), the molecule is saddle-shaped with only the carboxylic oxygen atoms pulled downwards out of the molecular plane towards the surface [56]. On Cu(111), the opposite is found. The oxygen atoms are pushed away from the surface, upwards out of the molecular plane, leading to a boat shape [35]. On hBN/Cu(111), the PTCDA molecule is nearly undistorted and hence planar [32]. It was surmised that the PTCDA molecule is bonded to the Ag(111) surface (and the KCl surface [57]) via the carboxylic oxygen atoms, while on Cu(111), the bonding proceeds primarily via the perylene core [58]. We hence suggest that the bonding via the perylene core on Cu(111) leads to a reduced flexibility of the intramolecular bonds of the core (including the central carbon ring), which causes the higher Raman shift of the RB mode. The flat, saddle-, and arch-like shapes of the molecule on hBN/Cu(111), Ag(111), and KCl/Ag(100) lead to smaller Raman shifts of the RB mode of 12 cm^−1^, 15 cm^−1^, and 25 cm^−1^, respectively. The special sensitivity of the RB mode to the interfacial bonding was also seen in high-resolution electron loss spectra [59].

We also compare with PTCDA multilayers. The Raman shifts of the multilayers was found at similar energies on all three substrates, that is, at 1,309.8 cm^−1^ for multilayers of PTCDA/hBN/Cu(111), at 1,309.5 cm^−1^ for 2.55 ML PTCDA/Cu(111), and at 1,309 cm^−1^ for 60 ML PTCDA/Ag(111) [53]. This is in agreement with an identical interaction between the PTCDA multilayers as it is expected. The energy of the RB mode for the multilayers is between that of the monolayer on hBN/Cu(111) (1,301.2 cm^−1^) and the monolayer on Cu(111) (1,312.9 cm^−1^). Actually, it is rather close to the value seen on Cu(111) within 3 cm^−1^ (see Table 1). This may indicate that intermolecular interactions between adjacent layers in a multilayer also have a significant impact on the vibrational properties and cannot be neglected. We note that these values are slightly larger than those measured for PTCDA single crystals [55] or thick films [43].

**2.2.2 Temperature induced changes in the Raman spectra:** The unique behavior of the RB mode can also be seen in its dependency on the preparation temperature both for PTCDA/hBN/Cu(111) and PTCDA/Cu(111). We compare spectra recorded directly after deposition at 20 K with those after annealing at 300 K (on hBN/Cu(111)) (Figure 3b) or with those after deposition at 300 K (on Cu(111)) (spectra not shown). For PTCDA deposited onto Cu(111) at 20 K, no Raman peaks could be observed at all in the sub-monolayer regime, and even for multilayers, the intensities of the Raman modes did not exceed two counts per second. (These are the modes given in Table 1.) At higher temperatures, the RB mode shifts to higher energies by 4 and 9 cm^−1^ for PTCDA on hBN/Cu(111) and Cu(111), respectively; the other modes remain unchanged (±0.4%, cf. Table 1).

This reflects the situation of PTCDA/Ag(111) [42] where the only significant temperature-dependent shift (by 10 cm^−1^) was also observed for the RB mode. In that study, the shift of the RB mode was originally attributed to be of chemical origin due to different bonding to the Ag(111) substrate present only in the monolayer but not for the multilayers. However, this interpretation appears less possible for the monolayers considered here. Hence, we recall a later study [56] that revealed the existence of a chemisorbed LT state of the monolayer in which the PTCDA molecule is positioned at a similar distance (±5%, regarding the perylene core) from the surface as the RT state but is significantly more distorted than the RT state (maximum height difference of atoms of 0.31 Å compared to 0.20 Å) [56]. In addition, the LT state is present as a disordered phase, while the RT state forms highly ordered domains [44]. This is accompanied by a change in the valence band structure as seen, for example, in UPS [44]. Thus, instead of a multilayer/monolayer effect, the above described differences in the Raman shifts may also be caused by the intermolecular interactions and a modification in the interfacial bonding in the ordered domains of the RT state, which form upon annealing at room temperature. We propose that, in particular, the effect of intermolecular interactions is also relevant for the monolayer of PTCDA on hBN/Cu(111), whereas on Cu(111) concomitant changes in the interfacial bonding may play a role, too. The increased temperature induces the formation of ordered domains (observed in LEED). The intermolecular interactions in the domains then cause a change in the structure and charge distribution within the molecule. This in turn increases the vibrational energies, in particular of the RB mode located on the perylene core.

#### 2.3 The role of surface defects for SERS

For both PTCDA/hBN/Cu(111) and PTCDA/Cu(111), the overall intensities of the Raman modes are of the same order, which implies that both surfaces cause a similar degree of Raman enhancement. However, we refrain from making a statement about the SERS effectiveness of hBN on Cu(111) for the following reason: In our experiments, the Raman intensities on either hBN/Cu(111) or Cu(111) were both found to be highly dependent on the exact position of the incident laser beam on the sample (varying by a factor of up to ca. 7). This indicates that the specific local surface quality, for example, the surface roughness at the spot where the Raman scattering occurred, influences the intensity of the Raman modes. Hence, we cannot draw quantitative conclusions here. Nevertheless, we gain some insight into this aspect from an experiment we conducted at low temperatures, which we describe in the following.

As mentioned in the previous section, in the sub-monolayer regime of PTCDA/Cu(111) deposited at 20 K no Raman peaks could be observed at all, and even for multilayers their intensities were very small (spectra not shown). For PTCDA on hBN/Cu(111) the situation is drastically different. Figure 3b shows the Raman modes of PTCDA (0.50 ML) after deposition at 20 K (orange) and after subsequent annealing at 300 K (blue). For both preparations, the Raman modes are clearly present and of a similar intensity.

We discuss two possible explanations for these different kinds of behavior after deposition at 20 K: (i) The dipole–dipole interaction between hBN and PTCDA enhances the Raman signal. In this process, the underlying copper would not be involved [50]. (ii) The Raman modes of PTCDA are strongly enhanced at specific adsorption sites, which we refer to as “hot spots”. On Cu(111), the molecules can reach these hot spots only by temperature-induced diffusion. Whereas, after deposition at 20 K, the molecules stay statistically distributed on the surface, and only a small fraction is located at these sites where the SERS effect occurs. On hBN/Cu(111), the significantly smaller interaction between PTCDA and the hBN surface compared to Cu(111) and a consequently smaller corrugation of the bonding potential lead to a much higher mobility, which allows for a diffusion to the hot spots even at low temperatures of 20 K.

We propose the second interpretation to be relevant. Our arguments are the following: Firstly, we have shown in a previous work [32] that the interaction between PTCDA and hBN/Cu(111) is of a physisorptive nature, which makes a strong SERS effect due to dipole–dipole interactions at the interface [50] unlikely. Secondly, the strong dependence of the Raman intensity on the sample position (see above) conforms with the interpretation of a SERS effect related to local hot spots. However, the details of the related adsorption sites remains unclear.

Since the SERS effect is primarily observed on rough/nanostructured rather than on flat metal surfaces [45], the SERS effect is expected to be larger for molecules located in proximity to surface defects. Thus, at surface positions with a higher defect density, the Raman intensities are expected to be higher. The relevance of the defect density in the present case is corroborated by our observation that at positions where the Raman intensity was high, the defect luminescence of the substrate (see region I in Figure 2) was also higher. In a theoretical study, García-Vidal and Pendry investigated the enhancement due to the SERS effect as a function of the roughness on a surface [48]. They found that on an inhomogeneously rough surface, the Raman spectrum is dominated by the enhanced signals from molecules located at features that exhibit a maximum in roughness. We can support this interpretation by results from a previous experiment. We observed by light microscopy that Ag(100) samples that had been prepared in a similar manner as the Cu(111) surface used here and that yielded a high-quality LEED pattern indicating the presence of long-range ordered and large, defect-free terraces, actually show variations in their surface morphology on a micrometer scale [60]. We propose that the defect-rich regions exhibit a large step density due to impurities and/or grain boundaries. Consequently, we assume that the hot spots are related to an inhomogeneous mesoscopic roughness of the Cu(111) surface, which is remnant after sputtering and annealing.

In conclusion, the Raman lines of PTCDA/hBN/Cu(111) and PTCDA/Cu(111) observed between 18,600 and 21,000 cm^−1^ are in accordance with the characteristic fingerprint of the molecule. They can be used as a monitor for its presence on the surfaces. Coverages cannot be determined quantitatively from the intensities of the Raman modes, since these depend on the sample positions, which we assign to a correlation with the local roughness due to structural defects of the surface. Note that we systematically did not observe the characteristic Raman line of hBN at approximately 1,370 cm^−1^ that was observed for hBN on SiO_2_/Si [61], on Cu foils [62], and on other metal foils [63–64] for our samples of hBN/Cu(111). This is an obvious discrepancy, which we cannot explain based on our current data. It may, however, be related to the specific interface between hBN and the single crystalline Cu substrate.

### 3 Fluorescence

#### 3.1 PTCDA/hBN/Cu(111)

We turn to the FL, which can be observed in region II of Figure 2, between 18,000 and 18,600 cm^−1^. We note ahead that we did not observe vibronic bands related to the FL peaks as it is the case, for example, for PTCDA on KCl [65].

**Figure 4:**
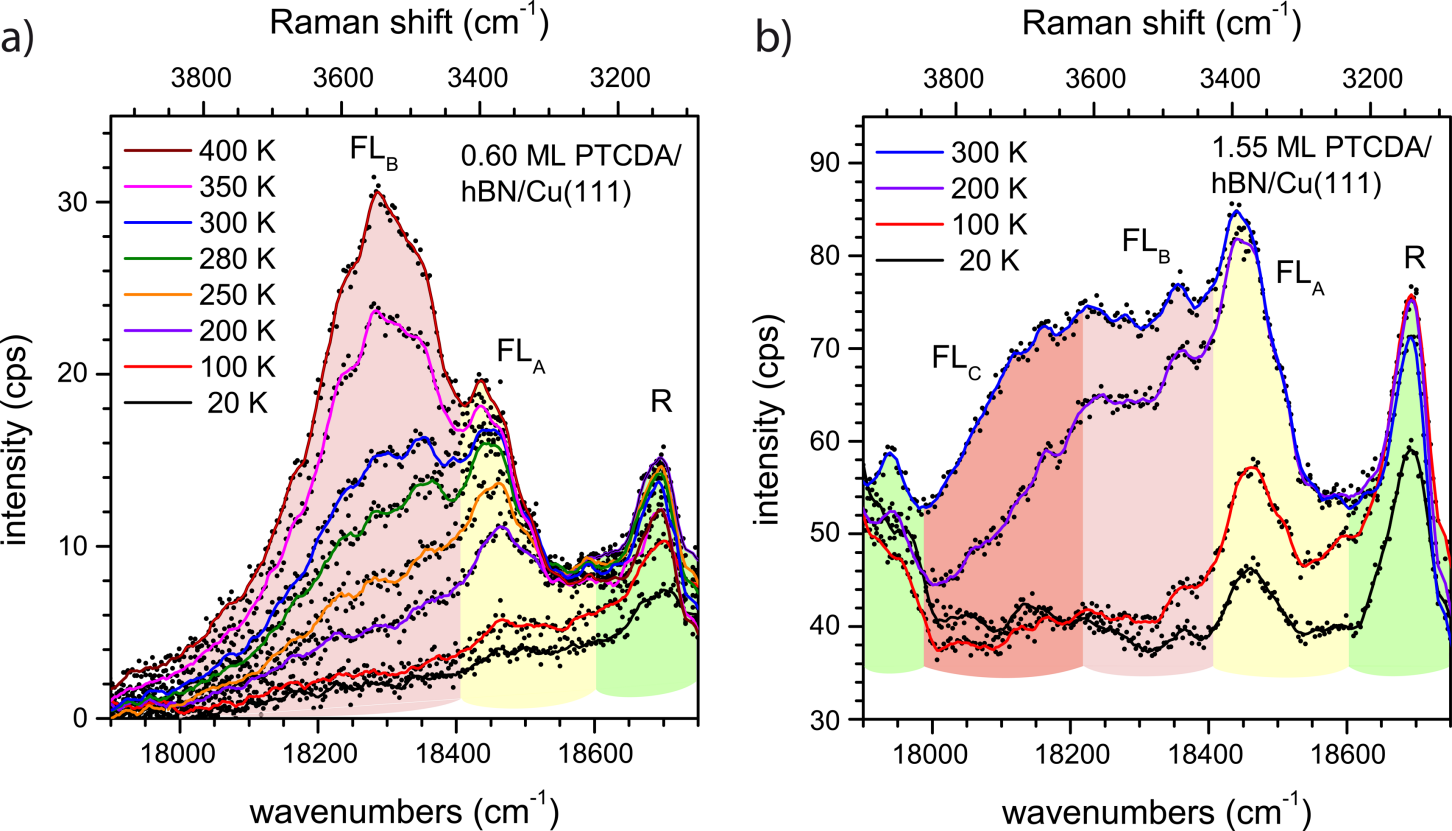
Fluorescence spectra of (a) 0.60 ML PTCDA and (b) 1.55 ML PTCDA on hBN/Cu(111) as deposited at 20 K (black) and subsequently annealed at different temperatures. Raman modes are highlighted in green, FL peaks FL_A_, FL_B_, and FL_C_ are highlighted in yellow, pink, and red, respectively. The spectrum of the clean surface was subtracted as a background. Spectra are smoothed (lines, original data as dots). All spectra were measured at 20 K using a grating with 600 grooves per millimeter.

First, we consider which experimental conditions led to the observation of FL peaks. Figure 4a shows a zoom-in on region II of the spectrum of 0.60 ML PTCDA on hBN/Cu(111) as deposited at a sample temperature of 20 K, and after a series of subsequent annealing steps at temperatures between 100 and 400 K. The peak on the high-energy side of the spectrum (marked in green and labeled “R”) is a Raman mode. This mode has also been observed for PTCDA/Ag(111) [53]. Furthermore, its change in intensity as a function of the annealing temperature agreed with that of other Raman modes. The behavior of the two other peaks depicted in Figure 4a (marked in yellow and pink) was quantitatively different. We assign these peaks to the FL of PTCDA molecules and will refer to them as FL_A_ (at ca. 18,450 cm^−1^) and FL_B_ (at ca. 18,300 cm^−1^). Both peaks appear after annealing at temperatures above ca. 200 K (FL_A_) and ca. 280 K (FL_B_).

We take this finding as an indication that the FL of PTCDA on hBN/Cu(111) depends on the structural order of the molecules on the surface that is established by annealing. We explain the existence of two FL peaks by the presence of two structurally different “phases” of PTCDA. Since all optical measurements were carried out at a sample temperature of 20 K, the temperature-induced structural ordering upon annealing that led to the FL peaks is irreversible. The broad FL peaks are superimposed with several sharp Raman lines, which lead to modulations of the peaks. There are two important differences between the FL_A_ and FL_B_ peaks: FL_B_ is significantly broader than FL_A_ (by a factor of ca. three, which we will discuss in detail in the Section Final Discussion). In addition, the intensity of FL_A_ saturates, while that of FL_B_ does not. Notably, the intensity of FL_A_ saturates upon annealing at 280 K, which is the temperature that is required for FL_B_ to be observed at all. This behavior was found for layers within a range of sub-monolayer coverages (between 0.10 and 0.80 ML).

For further insight, we refer to a temperature-dependent series of spectra at a higher coverage of 1.55 ML PTCDA on hBN/Cu(111), shown in Figure 4b. We observe the same Raman line as for the 0.60 ML spectrum, as well as FL_A_ and FL_B_. However, there are a few important differences compared to the spectra in Figure 4a: (i) FL_A_ can be observed in the spectrum immediately after deposition at 20 K; (ii) FL_B_ appears in the spectrum already after annealing at 200 K (not at 280 K); and (iii) additional FL intensity at lower energies (ca. 18,150 cm^−1^) appears, which we assign to a third peak FL_C_. The appearance of FL_A_ at low temperatures leads us to the conclusion that the attributed “phase A” of PTCDA/hBN/Cu(111) forms directly at higher coverages, while at lower coverages its formation requires annealing. Hence, we assign FL_A_ to PTCDA molecules at surface defects. In a sub-monolayer, the molecules can reach these defects via diffusion, which has to be temperature-induced. For a higher coverage, the sites at defects are already populated during the deposition, even at low temperatures. A saturation of the sites at defects leads to the observed intensity saturation of the corresponding FL_A_ peak. We note in this context that the surface defects connected to FL_A_ are not identical to the hot spots mentioned above, which support the SERS effect, as we will discuss below. After the saturation of the defects, the temperature-induced formation of a second “phase B” occurs leading to the peak FL_B_.

We also observed the FL_B_ peak on hBN/Cu(111) after deposition at 300 K for coverages of 1–3 ML. For all these experiments, we observed the LEED pattern that is characteristic of ordered domains of PTCDA/hBN/Cu(111) (see Appendix A). Thus, we conclude that FL_B_ stems from these ordered PTCDA domains in the first layer on hBN. The formation of ordered domains is not possible at a sample temperature of 20 K during deposition. It requires a certain threshold temperature (ca. 280 K). This explains why FL_B_ can only be observed after annealing or deposition at or above this temperature.

The energetic difference between FL_A_ and FL_B_ amounts to ca. 150 cm^−1^. We compare this to the energetic shift reported by Forker et al. who investigated PTCDA on hBN/Rh(111) [23]. They found that PTCDA molecules on hBN/Rh(111) are trapped in the pores of the hBN superstructure, which leads to isolated monomers on the surface. Annealing leads to the formation of ordered domains and a redshift of the spectrum by 223 cm^−1^ (27.6 meV) [23]. The molecules trapped in pores on hBN/Rh(111) can be compared with isolated molecules located at defects on hBN/Cu(111). However, please note that we do not consider trapped PTCDA molecules but isolated molecules to be the origin of FL_A_. This will be discussed in further detail in Section 3.3 in relation with further information from FL experiments. An energetic shift of the same order has also been observed for the FL of isolated molecules and that of ordered domains of PTCDA on NaCl [10]. Here, two different structures of ordered domains of PTCDA were observed (a herringbone and a quadratic structure) the S_0_/S_1_ transitions of which are redshifted relative to the isolated molecules by 560 cm^−1^ and 300 cm^−1^, respectively.

For a comparison of FL_B_ of PTCDA domains on hBN/Cu(111) at 18,300 cm^−1^, we refer to optical data taken for ordered monolayers of PTCDA on a monolayer of hBN grown on other substrates. The respective values are given in Table 2. Note that only PTCDA/hBN/SiO_2_ was investigated by FL spectroscopy while for PTCDA/hBN/Pt(111) and PTCDA/hBN/Rh(111) absorption spectra were measured. We cannot explain the differences of the S_0_/S_1_ transition energies, yet. However, we observe a trend of higher transition energies from hBN/SiO_2_ to hBN/Cu(111) to hBN/Pt(111) to hBN/Rh(111). This is the direction of increasing interactions between the hBN layer and the supporting metal substrate, as indicated by the increasing amplitude of the buckling of the hBN layers [30,66]. We note that Forker et al. [23] could exclude a dominant role of the dielectric properties of the metal substrates for the transition energies.

**Table 2:** Spectral positions of the S_0_/S_1_ transition of PTCDA domains in the first monolayer on hBN on SiO_2_ [22], Cu(111), Pt(111) [23], and Rh(111) [23]. PTCDA on hBN/SiO_2_ and on hBN/Cu(111) were investigated using FL spectroscopy. The results for PTCDA on hBN/Pt(111) and hBN/Rh(111) were gained in absorption experiments.

hBN/SiO_2_ [22]	hBN/Cu(111)	hBN/Pt(111) [23]	hBN/Rh(111) [23]

2.234 eV	2.26 eV	2.31 eV	2.38 eV
18,060 cm^−1^	18,300 cm^−1^	18,700 cm^−1^	19,200 cm^−1^

The peak FL_C_ (cf. Figure 4b) shows the same behavior as FL_B_ at a coverage of 1.55 ML. It is as broad as FL_B_ and appears after annealing at 200 K. However, it is not present at a coverage of 0.60 ML. Thus, it does not stem from PTCDA directly adsorbed on hBN, but from ordered PTCDA domains in a second layer. However, this is different from the FL emission (Y), which is present in bulk-like PTCDA layers and was reported, for example, in [67]. Indeed, we observed the broad Y line and its vibronic progression Y’ for higher coverages of 4–5 ML at 15,950 and 14,700 cm^−1^, respectively. This is in agreement with the line positions measured in [67] within 50 cm^−1^ and 300 cm^−1^, respectively.

In summary, for PTCDA/hBN/Cu(111) three FL peaks can be observed. FL_A_ (ca. 18,450 cm^−1^) is present in the first layer and stems from molecules at surface defects. To enable the molecules to reach these defects, a temperature-induced diffusion is necessary at sub-monolayer coverage. FL_B_ (ca. 18,300 cm^−1^) stems from ordered domains in the first PTCDA layer. It can only form under deposition at a sample temperature above ca. 280 K, or after annealing at this temperature. FL_C_ (ca. 18,150 cm^−1^) is assigned to the FL from ordered PTCDA domains in the second or higher layers.

#### 3.2 Comparison with PTCDA/Cu(111)

**Figure 5:**
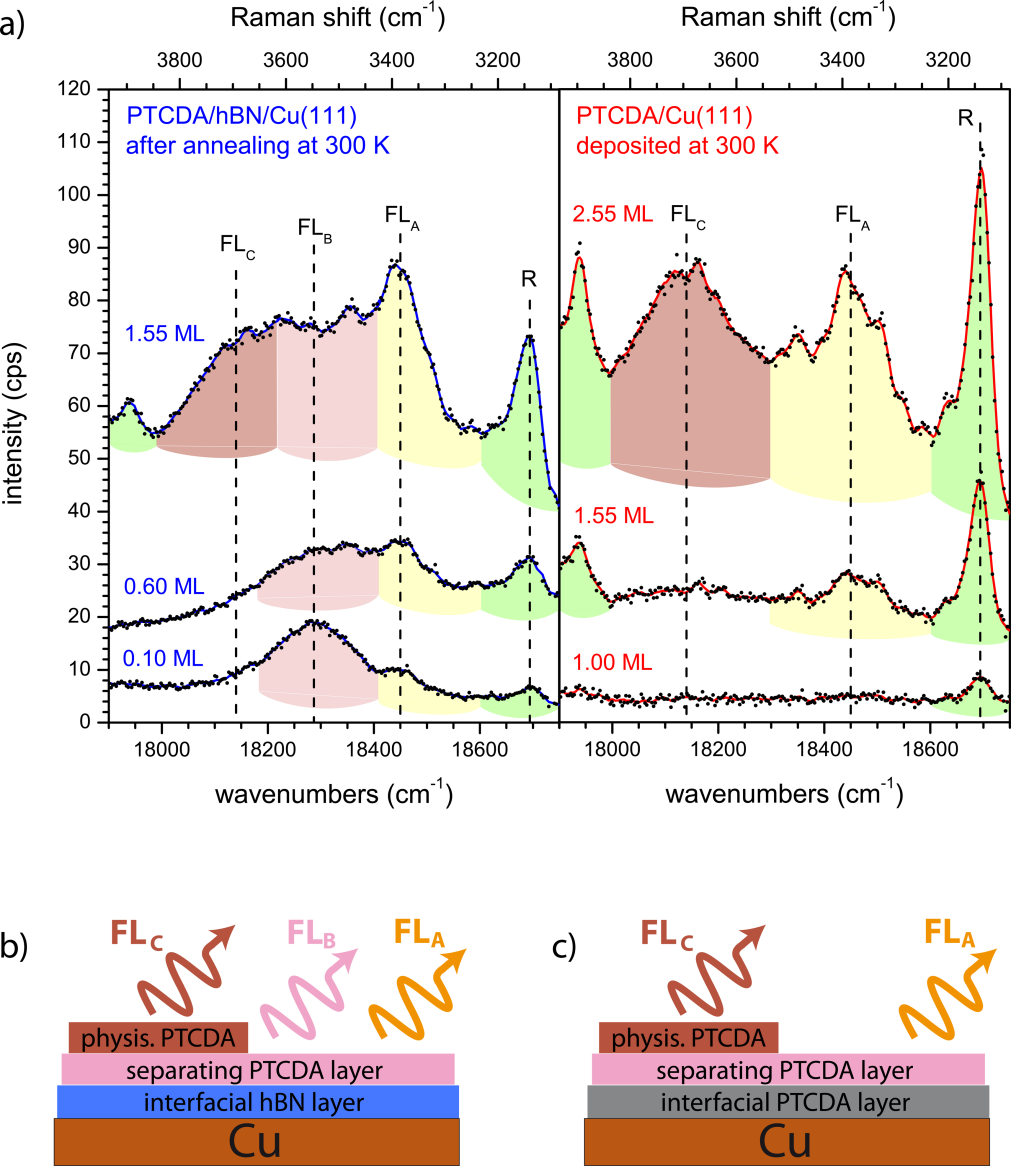
(a) Fluorescence spectra of PTCDA on hBN/Cu(111) (left, blue spectra, prepared by deposition at 20 K and subsequent annealing at 300 K) and PTCDA/Cu(111) (right, red spectra, prepared by deposition at 300 K). Raman intensities are highlighted in green; FL peaks FL_A_, FL_B_, and FL_C_ are highlighted in yellow, pink, and red, respectively. There is no FL from ordered domains in the first or second layers of PTCDA/Cu(111), but from the first layer of PTCDA/hBN/Cu(111). Spectra are smoothed (lines) and vertically shifted (original data as dots). Spectra of the clean substrates were subtracted as background. All spectra were measured at 20 K using a grating with 600 grooves per millimeter. (b) Schematic representations of 2 ML PTCDA/hBN/Cu(111) and (c) 3 ML PTCDA/Cu(111) and the FL signals observed from these layers. The FL from the first PTCDA layer on Cu is quenched (gray). The interfacial hBN layer (blue) and the interfacial chemisorbed PTCDA layer (gray) decouple the fluorescing molecules from the metal. Defect-mediated FL (FL_A_) is emitted from the separating PTCDA layer (pink) in both cases, FL_C_ originates from the physisorbed PTCDA layer (red). Only for PTCDA/hBN/Cu(111) FL occurs from PTCDA domains in the separating layer (FL_B_). For details on the different PTCDA layers, see text.

Figure 5a shows the FL spectra of PTCDA/hBN/Cu(111), prepared by deposition at 20 K and subsequent annealing at 300 K and of PTCDA/Cu(111), prepared by deposition at 300 K, both with varying coverages. We will list the important observations for these spectra: (i) There is no fluorescence at all for a full monolayer of PTCDA on bare Cu(111) while on hBN/Cu(111) FL can be observed for a coverage as low as 0.10 ML. (ii) In the second monolayer of PTCDA on bare Cu(111), an FL peak appears at 18,450 cm^−1^, which corresponds to FL_A_. (iii) In the third PTCDA layer on Cu(111), there is an FL peak at 18,150 cm^−1^, which is the position of FL_C_ observed for 1.55 ML PTCDA/hBN/Cu(111). (iv) FL_B_ is not observed at all for PTCDA/Cu(111). The absence of FL_B_ on Cu(111) is understood as we have assigned it to PTCDA domains on hBN/Cu(111). The other three observations have to be explained.

Ad (i): The absence of any FL of 1.00 ML PTCDA on Cu(111) confirms the complete quenching of the molecular excitation in the first layer on a metal surface as has previously been observed for the Ag(111) [5,39] and the Au(111) [39] surfaces. Ad (ii): For PTCDA/Cu(111), a sufficient decoupling to allow some FL is achieved for the second layer (1.55 ML). Thus, FL_A_ can be observed, which we assigned to FL from molecules at defects on the hBN/Cu(111) surface. It is remarkable that the PTCDA molecules have to interact with defects in order to fluoresce, and that the same kind of FL can be observed on both substrates. For PTCDA on Cu(111), as on hBN/Cu(111), a temperature-induced process is necessary to observe FL_A_ as PTCDA on Cu deposited at 20 K shows no FL at all (not shown). Only for PTCDA deposited at 300 K on Cu(111), we can observe the spectra shown in Figure 5. As stated in the previous section, we assume a temperature-induced diffusion process to surface defects where the PTCDA molecules are then able to fluoresce. The nature of these defects will be discussed in the next Section 3.3. Ad (iii): From our LEED experiments (see Appendix A) we know that PTCDA on Cu(111) grows layer-by-layer in the first three layers. Thus, we can assign the peak FL_C_, which appears in the third layer, to FL from ordered PTCDA domains adsorbed on top of two completed PTCDA layers. As we will discuss now, this is in accordance with the observation of FL_C_ from the second layer of PTCDA/hBN/Cu(111). We summarize our observations with the schematic layer models that are given in Figure 5b,c. In both cases an interfacial layer that is adsorbed on the Cu(111) surface exists. This layer decouples PTCDA molecules in higher layers, which leads to FL from these layers. The interfacial layer is either a layer of hBN or a monolayer of chemisorbed PTCDA. The next layer in both cases is formed by ordered PTCDA domains that show FL_A_, likely from molecules at defects. In the case of PTCDA on hBN/Cu(111), an additional FL_B_ from ordered domains is present. We call this first PTCDA layer a separating layer. In the TPD spectrum of PTCDA/hBN/Cu(111), the desorption peak of the separating layer can be distinguished from that of the multilayers (however, no separated peak for the second layer is observed) [32]. On Cu(111), the first (interfacial) layer does not desorb at all, and in the TPD spectrum, the second (separating) layer is also distinct from the multilayers [68]. The multilayers (both on hBN/Cu(111) and Cu(111)) are likely physisorbed and include the layers from the second and third layer onward. The ordered PTCDA domains in these layers are the origin of FL_C_.

Our findings are in accord with the conclusions by Stallberg et al. who measured photoluminescence spectra of PTCDA on Ag(111) and Au(111) [39]. From coverages of 1.8 ML on Ag(111) and 2.2 ML on Au(111) onward they observed FL peaks at 17,000 cm^−1^ and 17,300 cm^−1^ (denoted by M in [39]). These were assigned to PTCDA layers separated from the metal substrate by the first interfacial PTCDA layer. This M line was distinct from that of the bulk-like FL emission from thick films, which was found at 15,000 cm^−1^ (Y line) and 13,700 cm^−1^ (E line). The M line was observed even for multilayers, co-existing with the bulk emission. Similarly, for PTCDA/Cu(111), we observed the Y line and its vibronic progression Y’ of the multilayer FL for a coverage of 4.60 ML at 16,100 cm^−1^ and 14,750 cm^−1^, respectively, while FL_A_ and FL_C_ were still present (not shown). The Y line was assigned to the 0–0 transition of the PTCDA bulk phase [67]. Thus, the co-existence of the Y line and FL_A_ and FL_C_ is consistent with the formation of bulk-like clusters at a coverage of 4.60 ML. Likewise, on hBN/Cu(111), we observed the Y and Y’ lines for coverages from about 4–5 ML onward (see Section 3.1) in parallel with FL_A_, FL_B_, and FL_C_. This confirms, in accordance with our LEED data, that at least two complete layers of PTCDA form on both surfaces under the given preparation conditions.

#### 3.3 FL at defect positions

As stated above, we assign FL_A_ to molecules adsorbed at surface defects. Since FL_A_ can be observed for PTCDA/hBN/Cu(111) and PTCDA/Cu(111), we assume the same kind of defects has to be present on both surfaces. We propose that these defects are different to the metallic “hot spots” supporting SERS because FL_A_ is shifted in energy, which is less expected for coupling to metallic protrusions. An alternative origin of these defects could be the presence of carbon containing species due to segregation from the Cu bulk. Although this interpretation is speculative, we give some background in order to aid subsequent research. When the Cu(111) crystal was annealed at a temperature above 1050 K and for a period of time longer than 30 min, we observed an additional superstructure in LEED. Although we cannot identify these segregations unambiguously, we assume carbon segregation as carbon is known to form highly ordered nanostructures on Cu(111) [69]. Indeed, we adjusted our preparation by reducing the temperature to 1010 K during borazine deposition and omitting the annealing step under vacuum before borazine deposition to avoid this segregation (see Experimental section). However, we cannot exclude a small and randomly distributed residual amount of segregated carbon species since these would be invisible in LEED. We indeed found that samples showing segregated carbon in LEED do not allow for the formation of the hBN layer. Presumably, due to the extended carbon coverage, there is not enough bare Cu surface left for supporting the catalytic growth of hBN. Accordingly, on a surface with a small amount of carbon, growth defects in the hBN layer at positions where carbon is present may be expected. Thus, we consider carbon species in direct contact with the bare Cu that lead to similar or even identical defects on both surfaces (hBN/Cu(111) and bare Cu(111)), which, in turn, promote identical FL signals of PTCDA molecules. Note that these carbon-related defects are different from the defects that cause the SERS effect. The hot spots that lead to an enhancement of Raman signals are caused by structural defects of the Cu crystal, such as surface roughness, and not by a different chemical species.

Besides FL_A_, no further FL can be observed for 1.55 ML PTCDA/Cu(111) despite the fact that the LEED pattern shows well-resolved spots of ordered domains. The absence of FL from these ordered domains indicates that a single PTCDA monolayer on Cu(111) cannot decouple PTCDA in the second layer from the metal surface sufficiently enough for FL to occur. Thus, the observation of only FL_A_ for a coverage of 1.55 ML leads to the conclusion that the defects at which the fluorescing molecules are adsorbed also have a decoupling effect. This corroborates our deduction that the defects cannot be Cu ad-atoms.

We would like to reiterate that we exclude the possibility that the FL_A_ of PTCDA on hBN/Cu(111) stems from molecules trapped in the “moirons” of the hBN layer [24]. Studies on hBN/Cu(111) have shown a trapping of large organic molecules in the moirons of the electronic superstructure of hBN/Cu(111) at low molecular coverages [38]. At larger coverages, the entire surface was found to be homogeneously filled with molecules. Yet, they still showed site-dependent alterations in their electronic structure, namely a shift of the molecular frontier orbitals [38]. However, we propose that the situation of PTCDA on hBN/Cu(111) is different. First of all, as stated above, FL_A_ is also observed for PTCDA on Cu(111), which clearly excludes trapped PTCDA molecules on hBN as a possible origin for FL_A_. Furthermore, our own STM investigations showed no signs of a preferential occupation of the moirons on the hBN/Cu(111) surface by PTCDA [32]. Also, holes in the hBN layer that would allow for a direct contact between the molecules and the bare Cu(111) surface can be excluded as the origin for FL_A_ because the FL of PTCDA molecules in direct contact with the metal would be fully quenched.

### 4 Final discussion

We found that two layers of PTCDA are necessary to decouple PTCDA molecules from the Cu(111) surface in order to observe FL from ordered domains in the third layer. The same effect can be achieved by only one single layer of hBN. On hBN/Cu(111), two FL components (FL_A_ and FL_B_) are present for the first PTCDA layer, while a third one (FL_C_) can only be observed from the second layer onward. On Cu(111), the FL from the first PTCDA layer is completely quenched. FL_A_ and FL_C_ are observed only from the second and third layer onward, respectively. FL_A_ is assigned to a defect-related FL, while both FL_B_ and FL_C_ are assigned to ordered PTCDA domains. We can exclude bulk-like crystallites on top of the monolayer as the origin of the FL_B_ and FL_C_ peaks by the following arguments: (i) The LEED patterns of PTCDA on hBN/Cu(111) and on Cu(111) identify the formation of long-range ordered structures in the second layer. (ii) LEED investigations (see Appendix A) and TPD experiments [32,68] show that PTCDA/Cu(111) and PTCDA/hBN/Cu(111) form at least three or two complete layers, respectively. (iii) The spectral positions of FL_B_ and FL_C_ are unambiguously distinct from the Y and Y’ peaks of the FL of the PTCDA bulk.

We return to the question of CT across the hBN layer posed in the Introduction section. Our results indicate that the hBN layer is able to suppress the CT such that FL is observed. However, this suppression is not complete because the FL intensity is very low. In comparison to the FL of PTCDA observed on thin KCl films, we reckon that the FL on hBN/Cu(111) is smaller by a factor of ca. 10^4^ [70]. However, hBN should not be dismissed as a decoupling layer altogether. In an analogue experiment, we measured the FL spectrum of 5,10,15,20-tetraphenylbisbenz[5,6]indeno[1,2,3-*cd*:1’,2’,3’-*lm*]perylene (DBP) on hBN/Cu(111) (not shown). (For the chemical formula and optical properties of DBP, refer to the work by Rouillé et al. [71].) Here, we observed FL at sub-monolayer coverage [72]. The FL intensity was larger by a factor of ca. 10^2^ compared to the FL intensity of PTCDA on hBN/Cu(111). This can be explained by the fact that DBP is a lander-type molecule. It exhibits four peripheral phenyl groups, which function as spacers between the molecular backbone and the surface [71]. This presumably, in addition to the hBN layer and a weak interaction, supports the suppression of the CT process and allows for a higher FL intensity.

**Figure 6:**
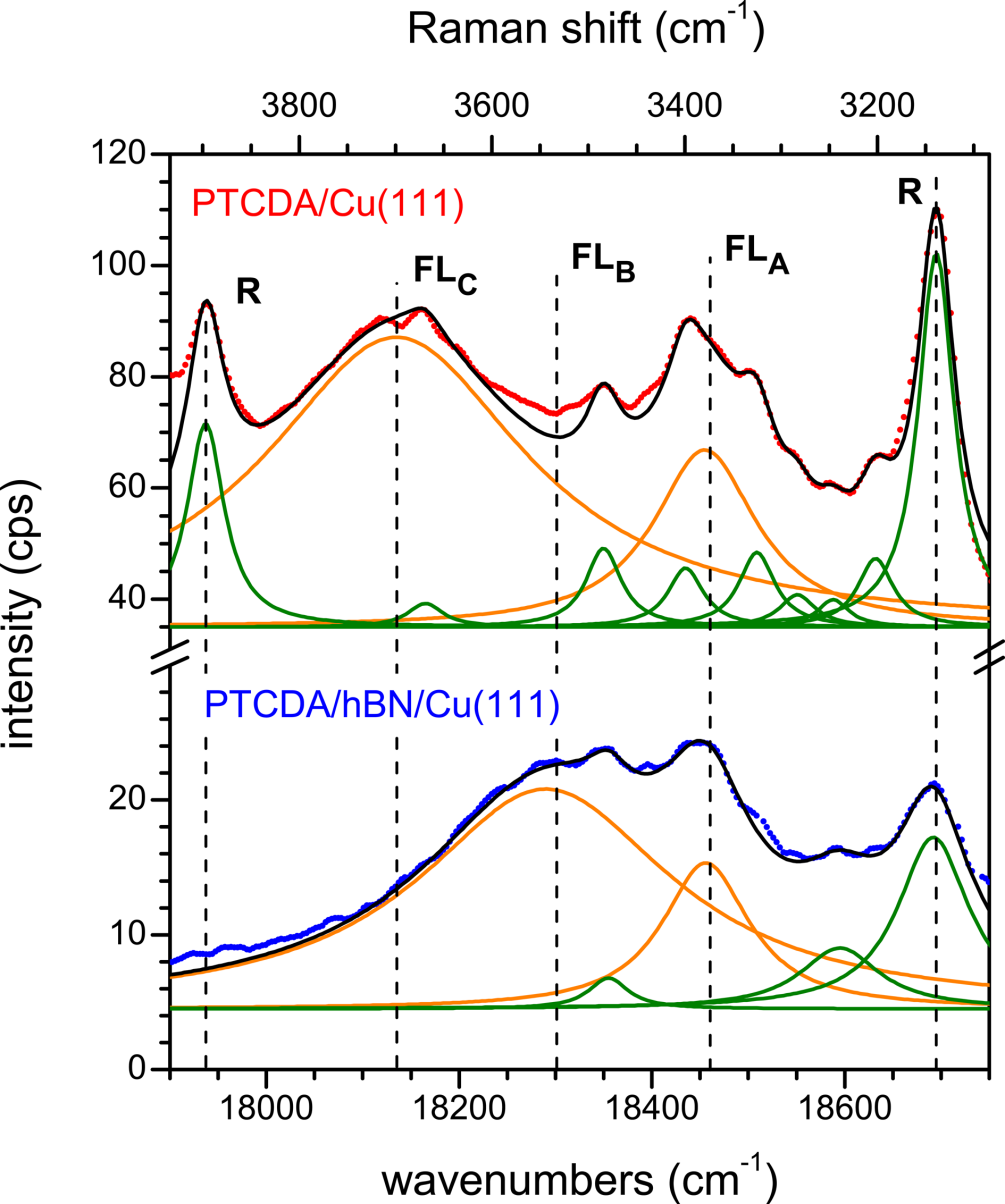
Fluorescence spectra of 0.60 ML PTCDA/hBN/Cu(111) (blue, bottom) and 2.55 ML PTCDA/Cu(111) (red, top). Original data (dots) are smoothed and shifted vertically. Both spectra were fitted using Lorentzian functions for the FL peaks (orange lines) and the most important Raman modes (green lines). Cumulative fits shown in black. Spectra of the clean substrates were subtracted as a background (cf. Figure 5). All spectra were measured at 20 K using a grating with 600 grooves per millimeter.

We further discuss the widths of the FL peaks on Cu(111) and hBN/Cu(111) in some detail. The large width of the FL peaks may partly be caused by disorder in the molecular domains or by a very small lifetime of the excited state. Since our LEED experiments showed the formation of ordered PTCDA domains, we assume that the main contribution for the line broadening is due to a reduced FL lifetime, τ_FL_, and that contributions from disorder and dephasing can be neglected [73–74]. Thus, the value of τ_FL_ measured from the full width at half maximum (FWHM) of the FL peaks on the frequency scale according to FWHM = 1/2πτ_FL_ yields a lower limit for the lifetime of the excited states. Because of the small FL yield we can assign τ_FL_ in good order to the time constant of the CT process. Hence, τ_FL_ gives quantitative information about the efficiency of the decoupling from the metal interface. Figure 6 representatively shows region II of the optical spectra of 0.60 ML PTCDA/hBN/Cu(111) and 2.55 ML PTCDA/Cu(111). Both spectra show FL_A_ and FL from ordered domains as either FL_B_ or FL_C_, respectively. The FL peaks and the Raman modes were fitted with sets of Lorentzians. The widths and positions of the narrower Raman lines were fixed during the fits. The fits were thus robust concerning the determination of the FWHM of the FL peaks. The lifetimes derived from these fits are given in Table 3. All of them are of a similar size and of the order of 10^−14^ s. Thus, we can understand why we did not observe final-state effects in the photoemission spectra (i.e., UPS) of PTCDA/hBN/Cu(111) due to CT across the interface, that is, the signature of uncoupled molecules [32], albeit the FL experiments demonstrate that some coupling to the underlying Cu(111) surface is present. The reason is that the time constant of this coupling is only of the same order as the time constant of the photoemission process. Therefore, it has only a small or negligible impact on the spectra. These small lifetimes are in agreement with the findings by Stallberg et al. [39], who showed that the lifetimes of the excited states of PTCDA in the second layer on Ag(111) and in the third layer on Au(111) are smaller than an upper value of 4 × 10^−12^ s, which was given by the time resolution for their experiment. The small lifetimes also explain why the FL intensities were so low with respect to those measured, for example, for an ordered monolayer of PTCDA/KCl/Ag(100) [65].

**Table 3:** Spectral positions of the FL peaks observed for PTCDA/hBN/Cu(111) and PTCDA/Cu(111), the species the FL peaks originate from, the FWHM of the FL peaks, and the lifetimes τ_FL_ of the respective excited states. Defect FL and physisorbed PTCDA apply for both PTCDA/hBN/Cu(111) and PTCDA/Cu(111).

	FL_A_	FL_B_	FL_C_

FL peak position	18,450 cm^−1^	18,300 cm^−1^	18,150 cm^−1^
assignment	defect related FL	PTCDA/hBN	physisorbed PTCDA
FWHM	(120 ± 10) cm^−1^	(320 ± 16) cm^−1^	(330 ± 15) cm^−1^
τ_FL_	(4.4 ± 0.3) × 10^−14^ s	(1.7 ± 0.1) × 10^−14^ s	(1.6 ± 0.1) × 10^−14^ s

Both the small FL intensities and the short lifetime of the excited state of PTCDA on hBN/Cu(111) show that, at least for this molecule, a single hBN layer is not sufficient to completely decouple the electronic states of the molecule and the metal. While the static CT related to the interfacial bonding is suppressed, as demonstrated by UPS [32], it is still significant for the excited state. A similar observation has been made for tetracene molecules on thin insulating layers of AlO*_x_* on Ni_3_Al(111) [75]. Here, too, the luminescence was found to be quenched despite the large bandgap of AlO*_x_* (6.5 eV) and the weak interactions at the interface due to a CT between overlapping π orbitals and the electronic states of the metal.

## Conclusion

We have measured the fluorescence of PTCDA on hBN/Cu(111) and Cu(111) to determine the efficiency of the electronic decoupling of PTCDA from the Cu substrate by a single hBN layer. The observation of Raman lines served as a monitor for the presence of PTCDA on the surface. In addition, LEED patterns show the formation of ordered structures and a layer-by-layer growth for at least the first two layers on hBN/Cu(111) and the first three layers on Cu(111).

The intensities of the Raman lines do not scale with the coverage, neither on hBN/Cu(111), nor on Cu(111). This clearly shows that additional aspects of the sample system, likely defects of the Cu substrate, play a role. We summarize the underlying mechanisms under the SERS effect. The small but discernibly different chemical shifts of Raman modes on the two surfaces are explained by the molecule–substrate interactions and specific bonding geometries of the molecule to the surface.

On both substrates, a broad fluorescence at ca. 18,450 cm^−1^ can be observed, which is identified as FL from molecules interacting with surface defects. This can be observed both for the first PTCDA layer on hBN/Cu(111) and the second layer of PTCDA on Cu(111). FL from the first PTCDA layer on Cu(111) is not observed. In contrast, the first PTCDA layer on hBN/Cu(111) already shows a weak FL from ordered domains at ca. 18,300 cm^−1^.

On bare Cu(111), two interfacial layers of PTCDA are required to achieve an equivalent decoupling of the third layer as one layer of hBN on Cu(111) for the first PTCDA layer. The third layer on Cu(111) shows FL at ca. 18,150 cm^−1^. This FL can be also observed in the second PTCDA layer on hBN/Cu(111) and is attributed in both cases to domains of physisorbed PTCDA on a separating PTCDA layer that forms on either the hBN layer or the first chemisorbed interfacial PTCDA monolayer on Cu(111).

While the charge transfer between PTCDA molecules and metal is sufficiently suppressed by a single hBN layer such that a weak fluorescence is observed, this suppression is limited. We estimate a lifetime of the excited state of ca. 10^−14^ s, which explains the very small fluorescence intensities. As a result, we find that a single layer of hBN on Cu(111) is able to decouple PTCDA molecules from the metal surface in so far as to prevent a total quenching of the fluorescence of the first molecular layer. However, a competing non-radiative channel for the decay of the excited state of the molecule remains as some charge transfer with a time constant of 10^−14^ s^−1^ is still possible. Thus, while a single layer of hBN provides a more efficient decoupling of a PTCDA layer from the Cu(111) surface than the first PTCDA layer itself, its efficiency is limited.

## Appendix A: Structural Investigations

Here, we present the structural investigations of monolayer PTCDA on hBN/Cu(111) and Cu(111) by LEED.

### The structure of PTCDA/Cu(111)

We begin with a summary of earlier reported results. At RT, PTCDA on the Cu(111) surface follows a layer-by-layer growth mode for the first three layers [76]. The obtained films remain unchanged under annealing at 530 K. From the fourth layer onward, the formation of nanocrystals begins. In TPD spectra, the desorption of the multilayer can be distinguished from the desorption of the second layer [68]. A desorption of the first layer was not observed due to a chemisorptive bonding to the Cu(111) surface. An STM study on the PTCDA monolayer on Cu(111) was published by Wagner et al. [77]. The authors reported two co-existing PTCDA phases, both displaying the characteristic herringbone arrangement, one commensurate, the other commensurate only in higher order.

In our experiments, PTCDA layers on Cu(111) were always grown at a sample temperature of 300 K. Experiments conducted in two different UHV chambers with different deposition rates resulted in identical LEED patterns. All LEED measurements were performed at 100 K.

**Figure 7:**
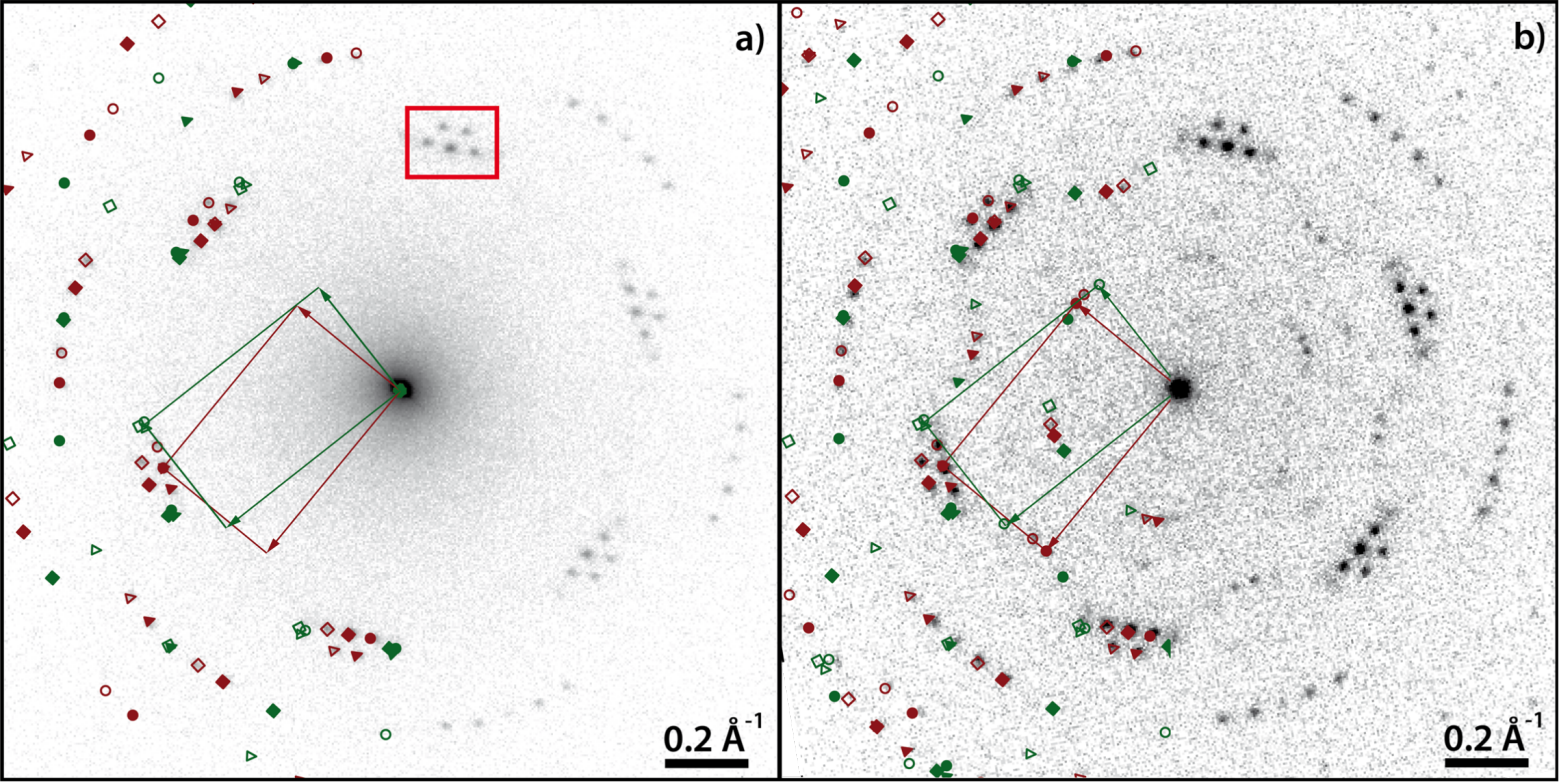
LEED pattern of (a) 1 ML PTCDA/Cu(111) and (b) 1.55 ML PTCDA/Cu(111) deposited at 300 K. On the left-hand side, the LEED patterns are superimposed with the corresponding simulations. Different colors refer to different PTCDA phases (dark red: α phase, green: β phase) and different symbols refer to different symmetry equivalent domains of a specific phase. The LEED patterns differ in the diffraction spots {*h*0} and {0*k*} for which *h* and *k* are odd. They are present in (b), but extinct in (a). The red box in (a) highlights the characteristic double-triangle arrangement of spots of the herringbone structure of PTCDA on surfaces. The LEED patterns were recorded at an electron energy of 77.6 eV and at a sample temperature of 100 K.

Figure 7a shows the LEED pattern of a complete monolayer PTCDA/Cu(111) with sharp diffraction spots. The spots marked in dark red are explained by one single phase (α). Additionally, a second phase (β, marked in green in Figure 7) is present. From the relative spot intensities, the α phase is considered as the majority phase. The monolayer LEED pattern is fully explained by the α and β phases with no systematic differences between the LEED pattern and the simulation. There are systematic extinctions of all {*h*0} and {0*k*} spots for which *h* and *k* are odd. This points to the presence of glide planes in both phases. The structural parameters, which can be determined from the LEED pattern with small margins of error (ca. 5%), are given in Table 4. The first columns of the superstructure matrices of the α phase and the β phase exhibit elements that are integer numbers (within the margins of error). This means that both phases exhibit on-line-coincidence (OLC) [78] with the Cu(111) surface.

**Table 4:** Structural results of PTCDA/Cu(111) according to the structure model proposed by Wagner et al. [77] (phase ‘1’ and ‘2’) and as determined from our LEED experiments (α phase and β phase) and of PTCDA/hBN/Cu(111). The vectors of the unit cells, **b**_1_ and **b**_2_, the angle β between the two, and the areas of the unit cells, *A*, are given. ϕ is the enclosed angle between the vector **b**_1_ and the substrate lattice vector, **a**_1_. ρ is the packing density of the molecules. Additionally, the superstructure matrices, **M**, are given. The matrix for PTCDA/hBN/Cu(111) refers to the Cu(111) surface at 100 K. We note that the parameters of phase ‘1’ and ‘2’ are derived from the (higher-order) commensurate structure models given in [77] and slightly deviate from the experimental STM results (within error margins). The error for β is the experimental error of the STM data. The matrices given for phase ‘1’ and ‘2’ differ from those reported by Wagner et al. [77] because they refer to a symmetry-equivalent domain according to the rules given in [79].

	Cu(111) - phase ‘1’ [77]	Cu(111) - phase ‘2’ [77]	

**b**_1_ (Å)	12.8(3)	13.4(1)	
**b**_2_ (Å)	22.1(3)	22.0(1)	
β (°)	90(5)	92(5)	
*A* (Å^2^)	283(10)	295(3)	
ϕ (°)	0	11	
ρ (molecules Å^−2^)	7.07 × 10^−3^	6.78 × 10^−3^	
**M**	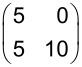	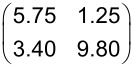	

	Cu(111) - α phase	Cu(111) - β phase	hBN/Cu(111)

**b**_1_ (Å)	12.4(2)	11.8(2)	12.07(3)
**b**_2_ (Å)	19.5(3)	19.9(5)	19.33(9)
β (°)	90.0(9)	90.0(13)	90(3)
*A* (Å^2^)	242(7)	235(10)	233(2)
ϕ (°)	2.5(7)	10.3(10)	9(1)
ρ (molecules Å^−2^)	8.26 × 10^−3^	8.51 × 10^−3^	8.85 × 10^−3^
**M**	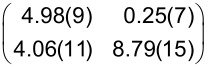	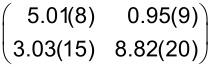	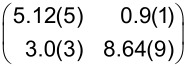

Both phases (α and β) exhibit a rectangular unit cell and belong to the *p*2*gg* space group. The differences between the two are the azimuthal orientations of the unit cell vectors relative to the Cu(111) surface vectors and the packing densities. The unit cell of the α phase is more closely aligned with the Cu surface vector **a**_1_ (2.5°) compared to the β phase (10.3°). Furthermore, the molecules in the β phase are slightly more densely packed than in the α phase (*A*_α_ = 103% × *A*_β_, cf. Table 4).

The most prominent feature of the LEED pattern is the characteristic arrangement of spots forming a “double triangle” (marked by a red box in Figure 7a). This is well known for the rectangular unit cell of PTCDA molecules forming the typical herringbone motif [80–81] that can also be found in the (102) plane of the bulk material [82]. In contrast to other coinage metal substrates, however, where two separate triangles consisting of six diffraction spots are observed [80,83]. Here, the {11} and {20} spots coincide, leading to two triangles that are characteristically corner-connected by one spot.

The PTCDA β phase on Cu(111) found in LEED bears significant similarity to the structure ‘2’ proposed by Wagner and co-workers [77]. Using the same rules for the matrix notations [79] for all structures shows that the matrix elements of structure ‘2’ and of the β phase found in our work differ only slightly (only one entry differs by more than 13%). The orientations of the unit cell vector **b**_1_ with respect to the substrate is identical for both structures within ±1.6° (cf. Table 4). Hence, we propose that the β phase possibly corresponds to structure ‘2’ by Wagner, although a difference in the size of the unit cell remains. Structure ‘1’ given by Wagner, however, was not observed in our LEED experiments and differs from our α phase. Furthermore, the packing densities found for the α phase and the β phase agree well (within 2.5%) with those of the (102) planes of the PTCDA bulk phases (8.40 × 10^−3^ and 8.32 × 10^−3^ molecules Å^−2^, respectively [82]), while the densities in structures ‘1’ and ‘2’ differ more significantly from these (by 14% and 21%). These differences may possibly be due to a difference in the substrate quality or preparation conditions in the two studies.

We now turn to PTCDA coverages above 1 ML. Both monolayer phases of PTCDA on Cu(111) (α and β) exist in parallel from the (sub-)monolayer to the multilayer regime. Additional deposition of PTCDA up to a coverage of 3 ML leads to additional weak spots in the LEED pattern (see Figure 7b). They can also be explained by the two PTCDA phases identified above. The spots that appear at distances of 0.3–0.6 Å^−1^ from the specular spot correspond to {*h*0} and {0*k*} spots of the α phase and the β phase for which *h* and *k* are odd and which were extinct before. No spot shifts as a function of the coverage were found. This may have two reasons. Either the molecules form only a single wetting layer on which additional molecules form clusters, or the higher layers adapt the same structure as the first layer without mismatch. Two observations point to the latter case: The intensity of the adsorbate diffraction spots increases with coverage while the relative intensity of the specular spot decreases. In addition, the {10} and {01} spots become detectable, which means that the glide planes of the adsorbate structure vanish. The glide lines in the space group *p*2*gg* of the PTCDA unit cell only exist if the underlying Cu(111) surface is not taken into account. For the first monolayer, this may be the case because the small periodicity of the Cu atoms compared to the PTCDA periodicity is not “felt” by the electrons scattered by a single PTCDA layer. For a second PTCDA layer, however, the periodicities in this layer and in the underlying monolayer are identical. Both layers are shifted laterally against each other [82], which breaks the glide plane symmetry. Thus, the LEED data indicate the growth of at least two or even more complete layers of PTCDA on Cu(111). This is in accordance with the results reported in [68,76].

In summary, our LEED investigations show that PTCDA on Cu(111) forms two co-existing phases, both of them displaying on-line-coincidence to the surface with a herringbone arrangement of the molecules. Both are observed from the (sub-)monolayer regime to at least the beginning of the growth of the third layer. The adsorbate forms at least two complete layers before clusters form.

### PTCDA on hBN/Cu(111)

**Figure 8:**
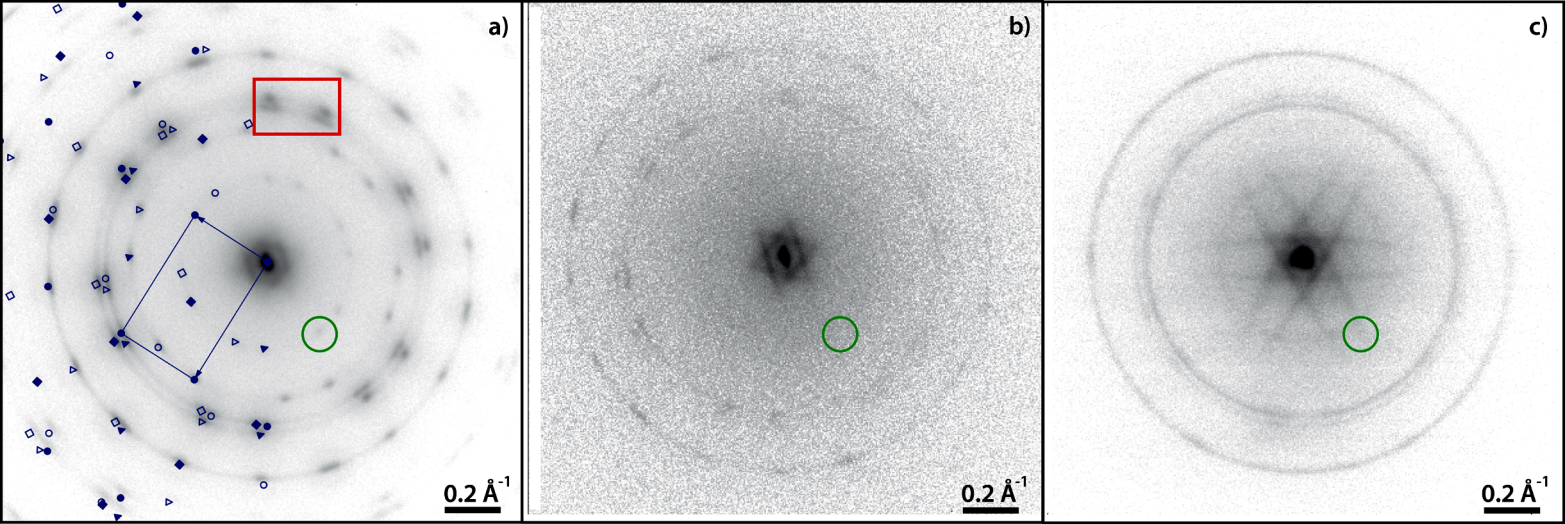
LEED pattern of PTCDA layers on hBN/Cu(111). (a) LEED pattern of 2 ± 0.5 ML PTCDA/hBN/Cu(111). The intensities of the LEED spots are azimuthally smeared out. The maxima in the ring intensities indicate the presence of azimuthally ordered domains. The left half of the LEED pattern is superimposed with the corresponding simulation. Different symbols refer to different symmetrically equivalent domains. This is the same LEED pattern as the one shown in [32], which we originally interpreted as a monolayer. More detailed analysis shows now that the second PTCDA layer has started to form (see main text). The red box highlights the characteristic double-triangle arrangement of spots of the herringbone structure of PTCDA on surfaces. The green ring marks a {10} diffraction spot. (b) LEED pattern of 0.8 ± 0.2 ML PTCDA/hBN/Cu(111). The LEED intensity is homogeneously distributed in rings around the specular spot. The radii of the rings match the distances of the spots in (a) from the specular spot. The green ring marks the same position as in (a). However, the rings corresponding to spot positions of {*h*0} and {0*k*} for which *h* or *k* are odd are extinct. (c) LEED pattern of 2 ± 0.5 ML PTCDA/hBN/Cu(111). The LEED intensity is homogeneously distributed in rings around the specular spot as in (b). At this higher coverage, the first-order PTCDA spots are no longer extinct. At the position of the green circle, a faint homogeneous ring can hence be observed. All LEED patterns were recorded at an electron energy of 31 ± 1 eV and at a sample temperature of 110 K.

Figure 8 shows LEED patterns of PTCDA/hBN/Cu(111) at a coverage of 2 ± 0.5 ML (Figure 8a,c) and 0.8 ± 0.2 ML (Figure 8b). All structures were grown at a sample temperature of 260 K and a deposition rate of 1 ML/min. They were observed in two different UHV chambers. LEED measurements were performed at 110 K. Note that the LEED pattern in Figure 8a has previously been published in [32]. In that study, we attributed this LEED pattern to a pure monolayer of PTCDA on hBN/Cu(111). Further analysis now leads us to the conclusion that the LEED pattern stems from a slightly higher coverage. This will be discussed in further detail below. Nonetheless, our findings presented in [32] still hold.

Commonly, two kinds of LEED patterns were observed for PTCDA/hBN/Cu(111) and were found from the sub-monolayer to the multilayer regime. The observable pattern, and thus the PTCDA structure that formed, did not depend on the PTCDA coverage, but rather on the quality of the hBN layer. The azimuthally smeared out intensity in Figure 8b,c (which was most commonly observed) points to an azimuthal disorder of the PTCDA domains on hBN/Cu(111), both in the first and in higher layers. In some cases, LEED patterns such as that given in Figure 8a were observed, which shows spots that are exceptionally not smeared out azimuthally. The radii of the rings in Figure 8b,c match the distances of the spots from the specular spot in Figure 8a.

We attribute the higher azimuthal order, which manifests in the more discrete spots, to a lower-quality hBN layer with defects or uncovered areas of Cu(111). Both may allow a pinning of PTCDA molecules to the Cu(111) surface. These molecules function as growth nuclei for PTCDA domains and determine the azimuthal orientation of these domains. Evidence for this interpretation is given by the fact that the orientation of the PTCDA domains on hBN/Cu(111) is in agreement with the orientation of PTCDA domains in the β phase on Cu(111) (within the error margins, cf. Table 4). The comparison to PTCDA/Cu(111) shows that the β phase has a higher structural resemblance with PTCDA/hBN/Cu(111) than the majority α phase, not only with regard to the orientation, but also to the size of the unit cell (with a deviation of 0.85% of the areas of the unit cells of PTCDA/hBN/Cu(111) and the β phase).

However, in general, PTCDA domains on hBN/Cu(111) have no intrinsically preferred orientation. In Figure 8b and Figure 8c, near the specular spot, a star-like pattern caused by multiple scattering of electrons on hBN/Cu(111) [30] can be observed. Its sharpness points to the high quality of the hBN layer that leads to the homogeneous distribution of the azimuthal orientation of the PTCDA domains.

The discrete spots in Figure 8a support the determination of the structural parameters (cf. Table 4). The similarity of this LEED pattern to those of other PTCDA structures on surfaces is obvious. This includes the double-triangle arrangement of spots (marked by a red box), which makes the structural difference of PTCDA on Cu(111) and on hBN/Cu(111) apparent. Here, the double triangle is formed by six spots leading to two separated triangles. (On Cu(111), these are connected at their corners.) The structure is incommensurate to the hBN layer, and also to the underlying Cu(111) surface. This is in accordance with a weak corrugation of the PTCDA/hBN interaction potential. The LEED pattern points to an arrangement of the molecules in the herringbone motif, as on Cu(111) [77] and other substrates [80,83]. This was also seen in STM measurements [32].

A comparison between the LEED pattern of the first PTCDA layer on hBN/Cu(111) (Figure 8b) with those of higher PTCDA layers (Figure 8a,c) shows that the diffraction spots {*h*0} and {0*k*} for which *h* or *k* are odd are extinct for the monolayer, but appear in the second layer. In Figure 8a, a discrete {10} spot is marked by a green circle. In Figure 8c, a line of a continuous ring that corresponds to the {10} spots of PTCDA is seen in the same circle. In Figure 8b, however, there is no intensity at this position in the LEED pattern. The same discussion on the extinction as for PTCDA/Cu(111) applies here (see Section “The structure of PTCDA/Cu(111)”). Furthermore, the distance of the diffraction spots from the specular spot does not change with increasing coverage. This indicates the same layer-by-layer growth mode of PTCDA on both Cu(111) and hBN/Cu(111).

In summary, monolayers of PTCDA/Cu(111) and PTCDA/hBN/Cu(111), despite their structural similarity, can be distinguished by their LEED patterns on the basis of two crucial features. On Cu(111), the “double triangle” is formed by five spots, making it a corner-connected double triangle, while on hBN/Cu(111) six spots form two distinct triangles. Furthermore, the azimuthal smearing of the spots of PTCDA on hBN/Cu(111) is specific. Additionally, LEED allows the identification of a second PTCDA layer on Cu(111) and hBN/Cu(111) due to the appearance of additional spots, which are absent in the monolayers for symmetry reasons.

## Appendix B: Additional optical spectra

**Figure 9:**
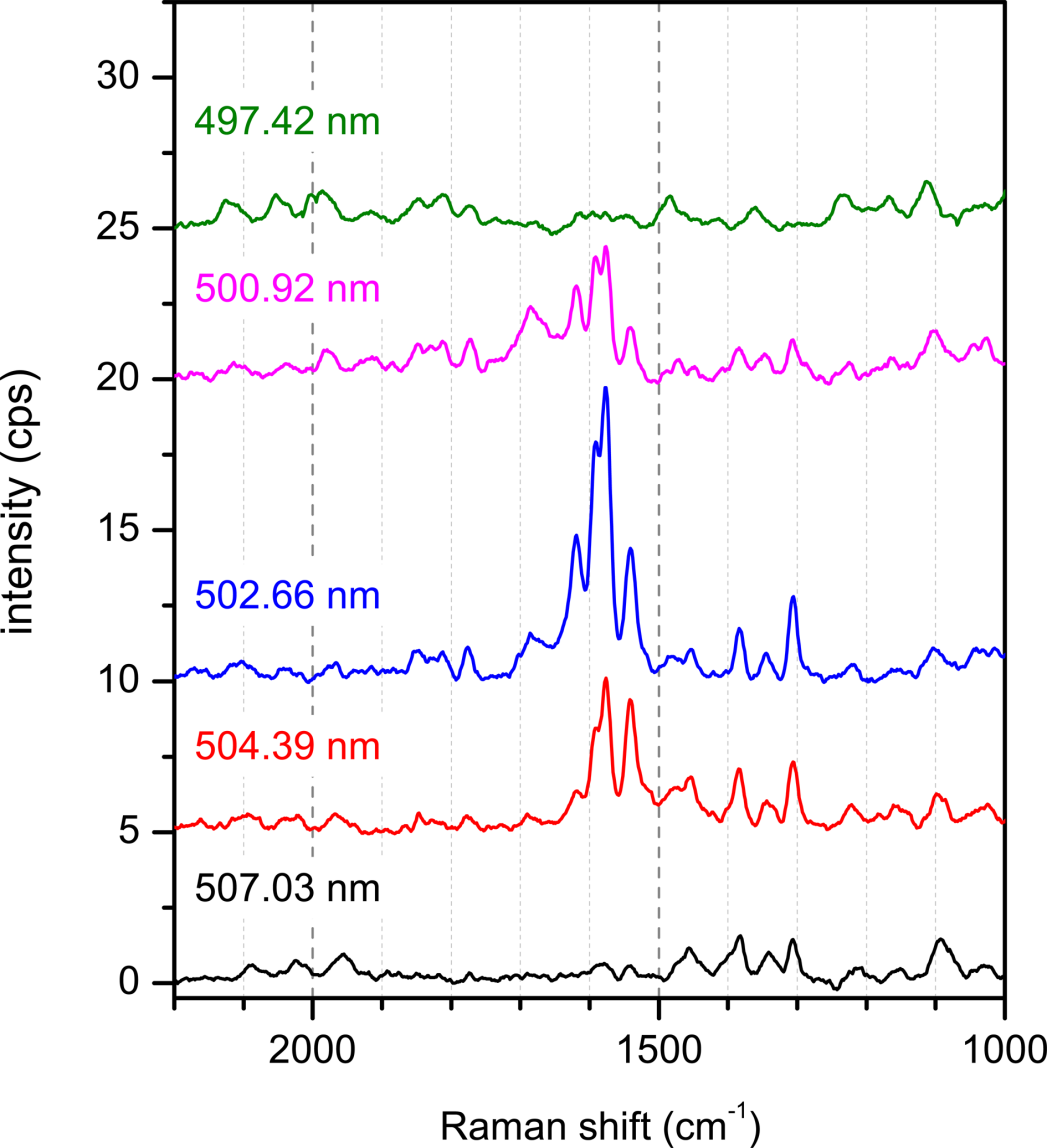
Raman modes of 0.90 ML PTCDA on hBN/Cu(111), measured with a dye laser with tunable wavelength (497–507 nm). Data are smoothed and shifted vertically after subtraction of a background. All spectra were measured at 20 K using a grating with 1200 grooves per millimeter.

Here, additional optical spectra that were referred to in the main text are shown. As mentioned in Section 3.1, the Raman lines in region III of the optical spectra were identified using a dye laser with a tunable wavelength. The Raman lines shift according to the wavelength of the laser. Figure 9 shows spectra recorded with wavelengths between 497 and 507 nm as a function of the Raman shift. Here, all Raman peaks stay at the same energies as expected. Notably, the intensities of the Raman modes change as a function of wavelength. The highest intensities are observed for 458 nm, exceeding the intensities for 502.66 nm (the highest in Figure 9) by a factor of seven.

**Figure 10:**
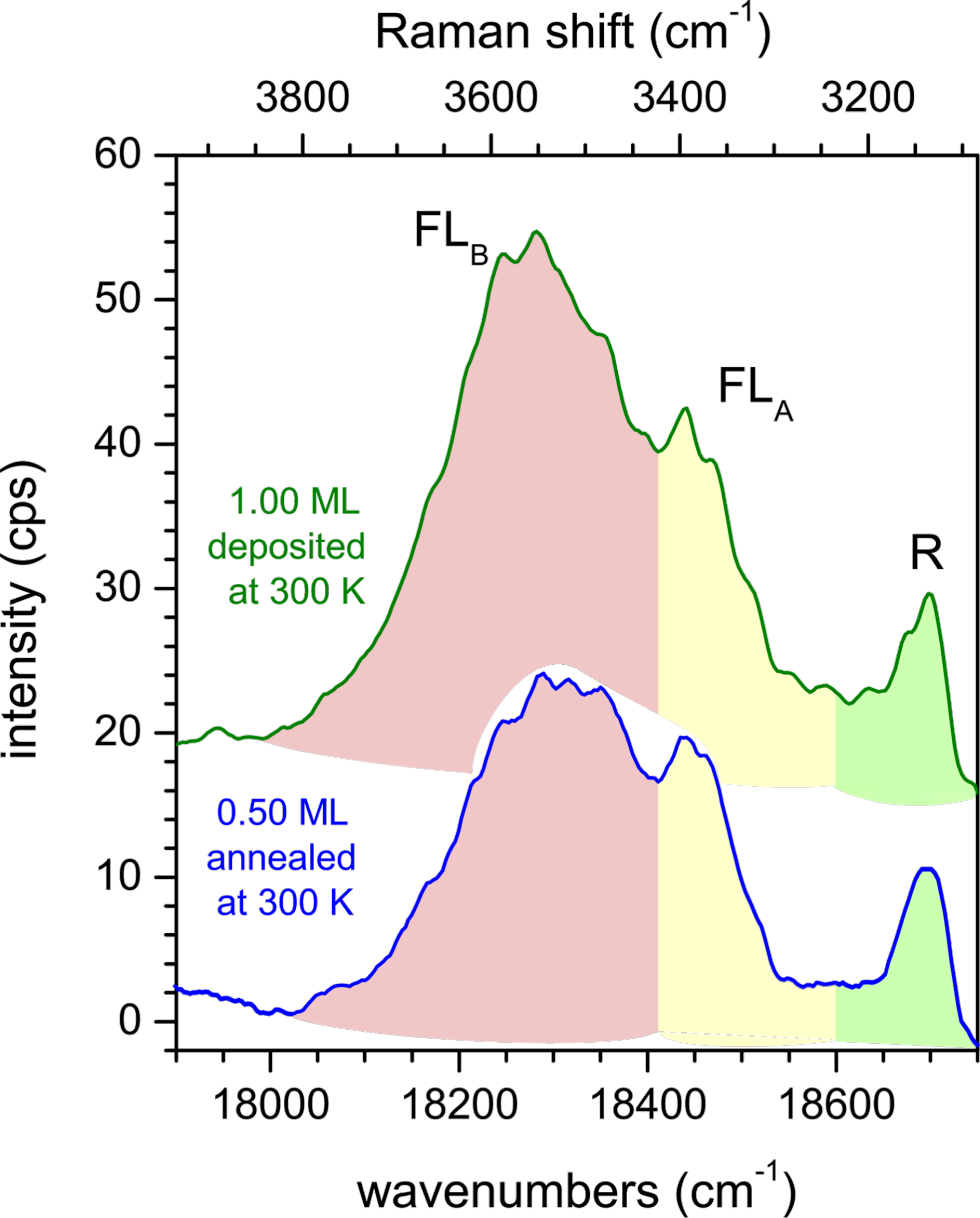
FL spectra of 0.50 ML PTCDA on hBN/Cu(111), deposited at 20 K and subsequently annealed at 300 K (blue, c.f Figure 2) and 1.00 ML PTCDA on hBN/Cu(111), deposited at 300 K (green). Data are smoothed and shifted vertically after subtraction of a background. All spectra were measured at 20 K using a grating with 600 grooves per millimeter.

In Section 3.1, we stated that PTCDA deposited on hBN/Cu(111) at a sample temperature of 300 K leads to the same FL as PTCDA deposited at 20 K and subsequently annealed at 300 K. Figure 10 shows spectra from PTCDA/hBN/Cu(111) prepared according to either recipe. In both cases, FL_A_ and FL_B_ are present.
